# The benefits of contrast-enhanced ultrasound in the differential diagnosis of suspicious breast lesions

**DOI:** 10.3389/fmed.2024.1511200

**Published:** 2024-12-24

**Authors:** Runa Liang, Jun Lian, Jinhui Zhang, Jiayu Jing, Jinxia Bian, Jinzhi Xu, Xin He, Shanshan Yu, Qi Zhou, Jue Jiang

**Affiliations:** ^1^Department of Ultrasound, The Second Affiliated Hospital of Xi’an Jiaotong University, Xi’an, China; ^2^Department of Ultrasound, Ankang Central Hospital, Ankang, China

**Keywords:** suspicious breast lesions, contrast-enhanced ultrasound, qualitative features, quantitative parameter, differential diagnosis

## Abstract

**Background:**

Contrast-enhanced ultrasound (CEUS) shows potential for the differential diagnosis of breast lesions in general, but its effectiveness remains unclear for the differential diagnosis of lesions highly suspicious for breast cancers.

**Objective:**

This study aimed to evaluate the diagnostic value of CEUS in differentiating pathological subtypes of suspicious breast lesions defined as category 4 of US-BI-RADS.

**Methods:**

The dataset of 150 breast lesions was prospectively collected from 150 patients who underwent routine ultrasound and CEUS examination and were highly suspected of having breast cancers. All lesions were pathologically confirmed by US-guided needle biopsy and surgery. The qualitative features and the quantitative parameters of CEUS of these breast lesions were analyzed. The CEUS and biopsy examinations were performed after informed consent.

**Results:**

In the qualitative features, crab clam-like enhancement, the presence of more than two enhanced vessels within lesions, and surrounding enriched vessels inserting into lesions were able to differentiate atypical fibroadenomas (FIB) and mass-like non-puerperal mastitis (NPM) from invasive ductal carcinomas (IDC) and ductal carcinomas *in situ* (DCIS) (*p* < 0.05). The enlarged scope, irregular shape, and perfusion deficiency were valuable to the differential diagnosis of FIB from the others (*p* < 0.05). In the four quantitative parameters of CEUS, only the peak intensity (IMAX) contributed to the differential diagnosis between malignant and benign tumors (*p* < 0.05, ROCAUC: 0.61, sensitivity: 60.4% and specificity: 65.9%, accuracy: 62.1%). However, IMAX did not show any difference in the paired comparison of IDC, DCIS, FIB, and NPM (*p* > 0.05). The logistic regression analysis results showed that heterogeneous perfusion, crab clam-like enhancement, and partial_ IMAX were independent risk factors for benign and malignant breast lesions *(p < 0.05)*. The area under a receiver operating characteristic of the integrated model was 0.89. In the diagnosis of benign and malignant pathological subtypes of breast lesions, independent risk factors and integrated models had no statistical significance in the diagnosis of IDC and DCISs, FIB, and NPM (*p* > 0.05).

**Conclusion:**

Some qualitative risk features of CEUS can distinguish malignant breast lesions from NPM and atypical FIB with a high score of US-BI-RADS, aiding physicians to reduce the misdiagnosis of suspicious breast lesions in clinical practice.

## Introduction

According to the statistical results of 36 kinds of cancers worldwide in 2022, the incidence rate of female breast cancer was ranked second, and the corresponding mortality rate was ranked fourth (1). Ultrasound (US) examination is an important and often-used tool to find breast lesions and distinguish the malignancies and benignities (2, 3). Currently, ultrasonographers predict the probabilities of malignant breast lesions according to the American Colleague Radiology US Breast Imaging—Reporting and Data System (ACR US-BI-RADS) (4). However, the diagnostic specificity of the high-risk categories remains widely controversial, especially for lesions scored as category 4 of US-BI-RADS, whose risk probability ranges from 2 to 95% (4), because of the highly overlapped risk features between malignant and benign breast lesions (5, 6). The technique of contrast-enhanced ultrasound (CEUS) can visualize the distribution and pattern of the microvascular environment within or surrounding organs or lesions (7–9), which has proven useful in differentiating malignant from benign breast lesions (10–13). However, the previous studies mainly evaluated the value of CEUS in differentiating benignity/malignancy of breast lesions overall (14–18), rarely focusing on the histopathological subtypes of breast lesions, especially for the atypical benignities that are easily mistaken for breast cancers.

Thus, in this study, we focused on the suspicious breast lesions defined as category 4 of US-BI-RADS and evaluated the diagnostic value of both qualitative features and quantitative parameters of CEUS in differentiating pathological subtypes of those lesions. To improve the accuracy of early diagnosis for such lesions, reduce unnecessary biopsy procedures, and obtain practical and highly accurate diagnostic guidelines for breast ultrasound contrast imaging, providing reliable and practical imaging diagnostic support for precise clinical diagnosis and treatment.

## Materials and methods

### Patients

**Figure 1:**
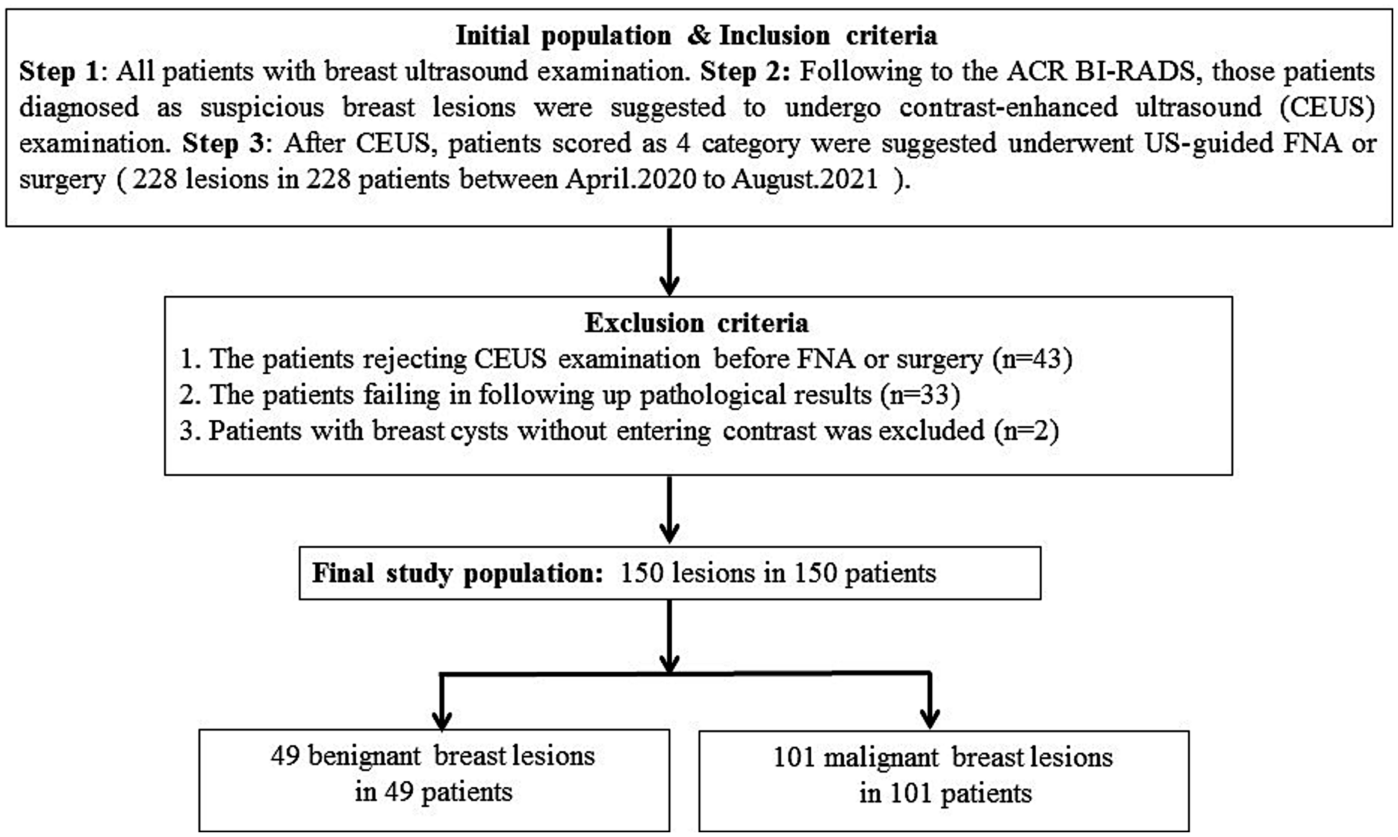
Flowchart of patients with breast lesions recruited in this prospective study.

In this prospective study, 228 single breast lesions from 228 patients were identified via the routine ultrasonic examination and classified as category 4 according to ACR US-BI-RADS (Figure 1). All patients were advised to undergo a CEUS examination before US-guided coarse needle biopsy (CNB) and surgery. However, 43 patients did not perform the CEUS examination and underwent surgery directly. A total of 33 patients failed to follow up and did not obtain their pathological results. Finally, 152 breast lesions from 152 patients got both histopathological results and CEUS videos successfully, and all the patients gave informed consent. The research institute granted ethical approval (No. 2018200) to carry out the study within its facilities. In the process of data analysis, two patients were excluded for failing to acquire high-quality CEUS data. Finally, 150 breast lesions from 150 patients were analyzed in this study (Figure 1). Of the 150 suspicious breast lesions, 101 (67.3%) were malignant (16 BI-RADS 4a, 31 BI-RADS 4b, 54 BI-RADS 4c) and 49 (32.7%) were benign (35 BI-RADS 4a, 12 BI-RADS 4b, 2 BI-RADS 4c) (Table 1).

**Table 1:** Histopathological subtypes of 150 suspicious breast lesions scored as category 4 of US-BI-RADS.

Histopathological subtype	Count (%)
Total (*n*)	150
Benign	49 (32.7%)
Atypical fibroadenoma Hyperplastic nodule Hyperplastic nodule with fibroadenoma	20 10 1
Intraductal papilloma	4
Mass-like non-puerperal mastitis	14
Mammary duct ectasia/periductal mastitis/ serous mastitis	7
Granulomatous lobular mastitis	7
Malignant	101 (67.3%)
Invasive ductal carcinoma	83
Ductal carcinoma in site	12
Invasive lobular carcinoma	2
Intraductal papillary carcinoma	2
Medullary carcinoma	1
Mucinous carcinoma	1

### Routine US and CEUS examination

Using the Siemens Acuson Sequoia 512 color Doppler ultrasound diagnostic system, the routine US examination was performed with an 18L6 linear transducer of frequency of 4.6–17.8 MHz, and CEUS was performed with a 10L4 linear transducer of frequency of 2.9–9.9 MHz. Patients were instructed to keep a supine position and expose the breast sufficiently. Ultrasonographers switched the routine model to the contrast model after finding a lesion. The contrast agent, 4.8 mL of SonoVue (Bracco Inc., Milan, Italy), was injected into the peripheral vein of the patient. The dynamic contrast-enhanced process within the lesion was observed and recorded for 2 min. The video data were stored automatically on the machine’s hard disk. Based on the grayscale images and the contrast-enhanced videos, we recorded the location, size, shape (regular/irregular), boundary (clear/unclear), blood types, echogenic foci (macro/micro-calcifications), and axillary lymph nodes of lesions. We also recorded the perfusion patterns and the direction of contrast entering the nodules, the enlarged size and the shape of enhanced lesions, and the enhanced vessels within and surrounding lesions. Finally, the DICOM format of the contrast videos was input into a quantitative software called TomTec SonoLiver v1.1.15.0, where the region of interest (ROI) of normal breast gland tissue and lesions (including the whole lesions and partial lesions with solid components) was marked and analyzed. The software provided five parameters based on its default smoothing curves, including: quality of fit (QOF, which measures the perfusion curve fitting degree), maximum intensity (IMAX, the echo intensity of the contrast medium at its peak), rising time (RT, the time from the onset of the contrast medium and its peak), time to peak (TTP, the time between the start of the contrast agent injection and its peak), and mean transit time (mTT, the mean local transition time of contrast media). The results of the software output in this study were completed by two highly trained radiologists. When the two radiologists disagreed, a third radiologist with over 10 years of experience made the final decision.

### Statistical analysis

The qualitative features were compared via the chi-square and Fisher’s exact test. The quantitative parameters were compared via a *t*-test, Bartlett’s test, ANOVA test, and Kruskal test. A *p* < 0.05 was considered statistically significant. Single-factor and multi-factor logistic regression with forward stepwise analysis were applied to screen for independent risk factors and establish an integrated model to identify benign and malignant lesions as well as histopathological subtypes of suspicious breast lesions. The ROC curve was used to evaluate the integrated model and calculate the area under the curve (AUC). The cutoffs for the qualitative features and quantitative parameters of CEUS were determined, and the corresponding sensitivity and specificity were calculated based on the maximum value of Youden’s index. The DeLong test was used to compare the differences between the AUCs of the ROC curves. All statistical analyses were executed using R software version 4.0 (R codes are shown in the Supplementary material).

## Results

### Patient information

Of 150 breast lesions scored as category 4 of US-BI-RADS, 101 were breast cancers (16 BI-RADS 4a, 31 BI-RADS 4b, and 54 BI-RADS 4c) and 49 were atypical benign lesions (35 BI-RADS 4a, 12 BI-RADS 4b, and 2 BI-RADS 4c). The average age of the patients with malignancies and benignities was 52.8 and 44.6, with standard deviations of 13.5 and 10.9, respectively. The 49 benign lesions included 20 fibroadenomas (FIB) with atypical grayscale ultrasonic features, 10 hyperplastic nodules, 1 hyperplastic nodule with FIB, 4 intraductal papillomas, and 14 mass-like non-puerperal mastitis (NPM). The 101 malignant lesions included 83 invasive ductal carcinomas (IDC), 12 ductal carcinomas in site (DCIS), 2 invasive lobular carcinomas, 2 intraductal papillary carcinomas, and one mucinous carcinoma (Table 1). The average size of 49 benign lesions is 18.2 mm with a standard deviation of 12.1 mm, and the average size of 101 malignant lesions is 23.2 mm with a standard deviation of 13.3 mm (Supplementary Table 1). Other data, including age, menopausal history, family history, location of lesions, lymphatic metastasis, and ultrasound and contrast-enhanced ultrasound characteristics, are shown in Supplementary Table 1.

### Qualitative features of CEUS in differentiating the histopathological subtypes of suspicious breast lesions

Among the risk qualitative features of CEUS, enlarged scope, heterogeneous perfusion, perfusion deficiency, crab clam-like enhancement, more than two enhanced vessels within lesions, and surrounding enriched vessels inserting into lesions represented statistically significant differences for differentiating benign from malignant lesions (*p* < 0.01). Of these features, crab clam-like enhancement had the highest specificity of 95.9%, but the lowest sensitivity of 51.5%, with moderate accuracy of 66.0% (Table 2). The surrounding enriched vessels inserting into lesions had the highest sensitivity of 100% and the highest accuracy of 87.3%, though with moderate specificity of 61.2% (Table 2).

**Table 2:** Diagnostic performance of qualitative risk features of CEUS in 150 suspicious breast lesions.

Risk features of enhanced lesions	Benign (*n* = 49, yes/no)	Malignant (*n* = 101, yes/no)	SEN (%)	SPE (%)	ACC (%)	*P*-value^a^
Enlarged scope	18/31	67/34	66.3	63.3	65.3	<0.01
Irregular shape	26/23	71/30	70.3	46.9	62.7	0.06
Directed perfusion	25/24	52/49	51.5	49.0	50.7	1.00
Heterogeneous perfusion	20/29	81/20	80.2	59.2	73.3	<0.01
Perfusion deficiency	20/29	78/23	77.2	59.2	71.3	<0.01
Crab clam-like enhancement	2/47	52/49	51.5	95.9	66.0	<0.01
More than two enhanced vessels within lesions	5/44	61/40	60.4	89.8	70.0	<0.01
Surrounding enriched vessels inserting into lesions	19/30	101/0	100	61.2	87.3	<0.01

^aChi-square test. SEN, sensitivity; SPE, specificity; ACC, accuracy.^

**Figure 2:**
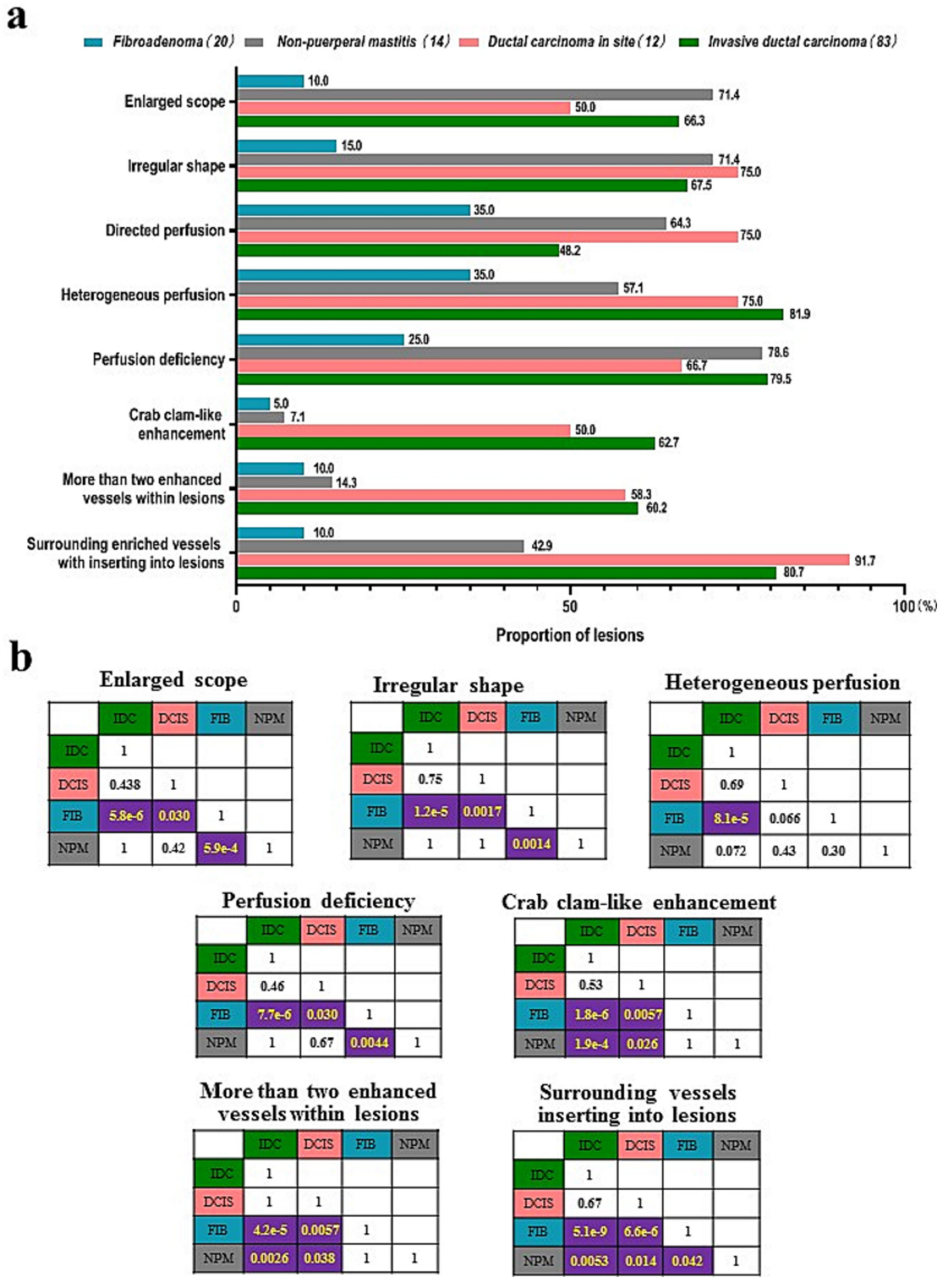
Comparison of the qualitative features risk features of CUES among four histopathological subtypes of breast lesions. **(A)** Proportion of the qualitative risk features. **(B)** *p*-values of the paired comparisons of four histopathological subtypes. IDC, invasive ductal carcinoma; DCIS, ductal carcinoma in site; FIB, fibroadenoma; NPM, non-puerperal mastitis.

**Figure 3:**
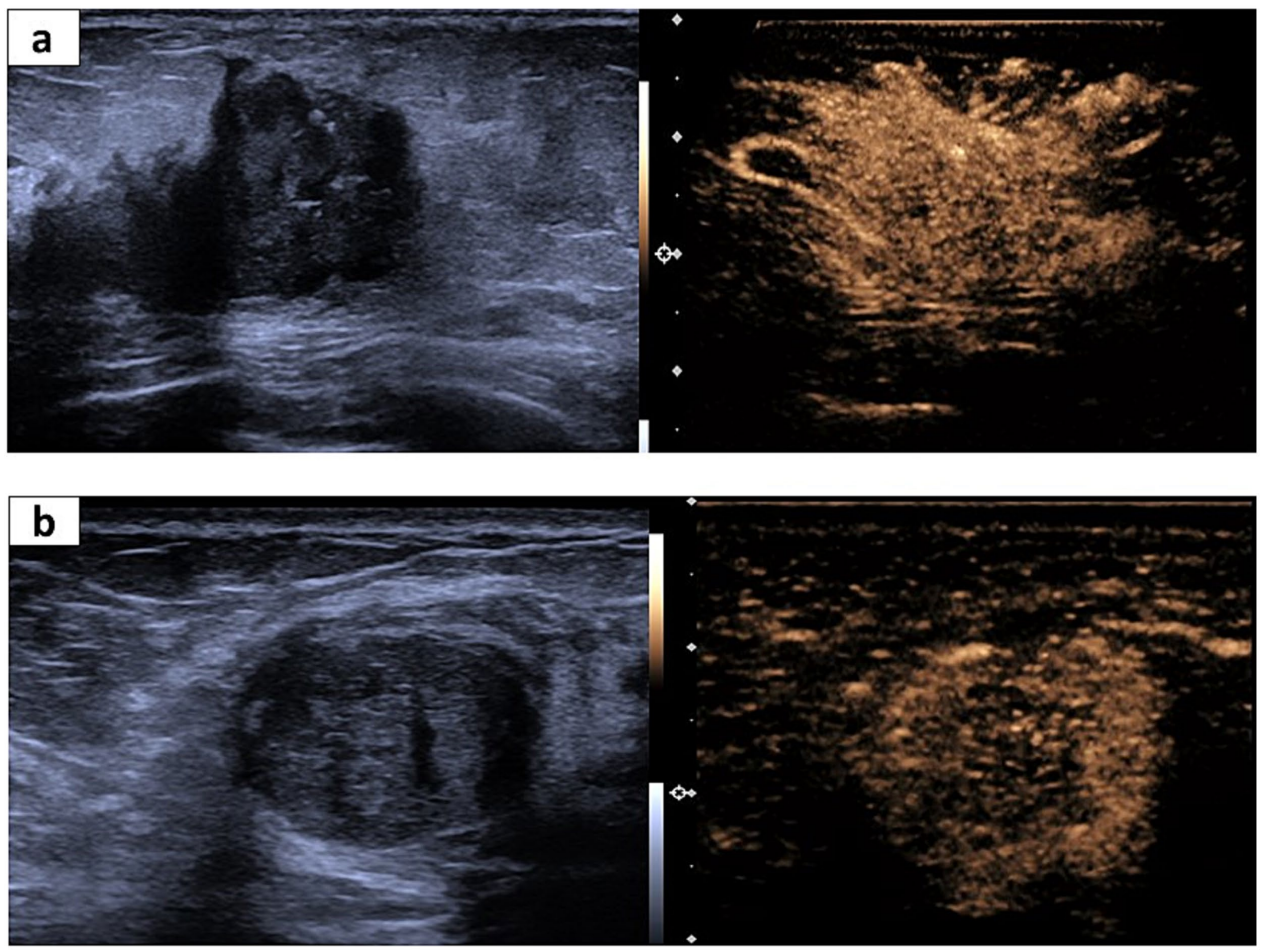
Representative US and CEUS images for invasive ductal cancer (IDC) and fibroadenoma. **(A)** A 65-year-old woman suffering from IDC. The irregular shape and partially unclear margin in the US image (left). The corresponding CEUS image shows surrounding enriched vessels inserting into the lesion, clam-like enhancement, and more than two numbers of enhanced vessels within the lesion (right). **(B)** A 56-year-old woman suffering from fibroadenoma. Regular shape and a partially unclear margin in the US image (left). The corresponding CEUS image shows surrounding enriched vessels paralleled to the lesion and <2 numbers of enhanced vessels within the lesion.

In a comparison of CEUS qualitative features for differentiating the four histopathological subtypes (IDC, DCIS, FIB, and NPM), except the directional perfusion, other risk features showed significant overall differences (*p* < 0.01) (Supplementary Table 2). In the paired comparison, the crab clam-like enhancement, more than two enhanced vessels within lesions, and surrounding enriched vessels inserting into lesions could differentiate IDC and DCIS from atypical FIB and NPM (*p* < 0.05) (Figures 2, 3) but showed no significant difference between IDC and DCIS, or between FIB and NPM (*p* > 0.05) (Figure 2). The enlarged scope, irregular shape, and perfusion deficiency were valuable in differentiating FIBs from the others (*p* < 0.05), although they showed no significant difference among the paired comparison of IDC, DCIS, and mass-like NPM (*p* > 0.05) (Figure 2). The heterogeneous perfusion showed a significant difference only between IDC and atypical FIB (*p* < 0.05) (Figure 2).

### Quantitative features of CEUS in differentiating the histopathological subtypes of suspicious breast lesions

After the quantitative analysis, 140 of 150 breast lesions had QOFs over 50% and the QOFs values were higher in the whole enhanced lesions than in the normal enhanced breast gland tissue and partially enhanced lesions with solid composition (Supplementary Figure 1). In a comparison of four quantitative parameters (IMAX, RT, TTP, and mTT) of CEUS in differentiating the benign from malignant breast lesions, only IMAX was significantly higher for the malignancy than the benignity in both whole and part of enhanced lesions (*p* < 0.05). The other three parameters showed no significant difference between malignancies and benignities (Supplementary Figure 2 and Supplementary Table 2). IMAX showed the highest diagnostic value for differentiating the benign from malignant breast lesions for the whole lesions (AUCROC: 0.62, 95% confidence interval: (0.52: 0.72), the cutoff value of 299.4%, sensitivity: 44.8%, specificity: 77.3%, negative predictive value: 39.1%, positive predictive value: 81.1%, accuracy: 55%, *p* < 0.05), and the partial lesions with solid components (AUCROC: 0.61, 95% confidence interval: (0.51: 0.71), cutoff value: 695.6%, sensitivity: 60.4%, specificity: 65.9%, negative predict value: 43.3%, positive predict value: 79.5%, accuracy: 62.1%, *p* < 0.05) (Table 3 and Supplementary Figure 2). In the paired comparison of the histopathological subtypes of breast lesions, none of the quantitative metrics showed a significant difference in differentiating between IDCs, DCISs, FIBs, and NPMs (*p* > 0.05) (Supplementary Figure 3 and Supplementary Table 4).

**Table 3:** Comparison of four quantitative parameters of CEUS in differentiating atypical benignities from malignant breast lesions.

Quantitative parameters	SEN (%)	SPE (%)	ACC (%)	NPV (%)	PPV (%)	AUROC (%) (95% CI)	Cutoff value
Whole lesion							
Whole_IMAX	44.8	77.3	55.0	39.1	81.1	0.62 (0.52, 0.72)	299.4%
Whole _RT	59.4	54.6	57.9	38.1	74.0	0.56 (0.46, 0.67)	9.0 s
Whole _TTP	92.7	18.2	69.3	53.3	71.2	0.51 (0.41, 0.62)	16.9 s
Whole _mTT	76.0	45.5	66.4	46.5	75.3	0.61 (0.50, 0.71)	29.6 s
Partial lesion							
Partial_IMAX	60.4	65.9	62.1	43.3	79.5	0.61 (0.51, 0.71)	267.3%
Partial _RT	57.3	52.3	55.7	35.9	72.4	0.54 (0.43, 0.64)	7.7 s
Partial _TTP	54.2	59.1	52.1	37.1	74.3	0.52 (0.41, 0.63)	10.7 s
Partial _mTT	88.5	27.3	55.7	52.2	72.6	0.56 (0.45, 0.66)	31.2 s

AUROC, area under receiver operating curve; CI, confidence interval; SEN, sensitivity; SPE, specificity; PPV, positive predictive value; NPV, negative predictive value; IMAX, maximum intensity; RT, rising time; TTP, time to peak; mTT, mean transit time.

### Logistic regression analysis of qualitative and quantitative features of CEUS in differentiating the histopathological subtypes of suspicious breast lesions

In the establishment of the logistic regression analysis model, heterogeneous perfusion, crab clam-like enhancement, and partial_IMAX were independent risk factors for benign and malignant breast lesions (*p* < 0.05). Of these features, the crab clam-like enhancement had the highest OR of 30.91. Heterogeneous perfusion and partial_ IMAX OR values are 5.46 and 1.01, respectively (Table 4).

**Table 4:** Logistic regression analysis of CEUS features.

Variables	*β*	S.E	Z	*P*	OR (95% CI)	*β*	S.E	Z	*P*	OR (95% CI)
Enlarged scope										
0					1.00 (Ref)					
1	1.57	0.38	4.08	**<0.001**	4.79 (2.26–10.16)					
Irregular shape										
0					1.00 (Ref)					
1	1.01	0.36	2.82	**0.005**	2.75 (1.36–5.54)					
Heterogeneous perfusion										
0					1.00 (Ref)					1.00 (Ref)
1	1.77	0.38	4.62	**<0.001**	5.87 (2.77–12.44)	1.70	0.48	3.54	**<0.001**	5.46 (2.13–14.00)
Perfusion deficiency										
0					1.00 (Ref)					
1	1.59	0.38	4.25	**<0.001**	4.92 (2.36–10.26)					
Crab clam-like enhancement										
0					1.00 (Ref)					1.00 (Ref)
1	3.62	0.75	4.83	**<0.001**	37.36 (8.58–162.59)	3.43	0.77	4.43	**<0.001**	30.91 (6.78–140.83)
More than two enhanced vessels within lesions										
0					1.00 (Ref)					
1	2.60	0.51	5.05	**<0.011**	13.42 (4.90–36.75)					
Whole_ IMAX	0.01	0.00	2.53	**0.011**	1.01 (1.01–1.01)					
Partial_ IMAX	0.01	0.00	2.68	**0.007**	1.01 (1.01–1.01)	0.01	0.00	2.14	**0.032**	1.01 (1.01–1.01)

OR, odds ratio; CI, Confidence interval; Ref, reference. The bold value is *p* < 0.05.

**Figure 4:**
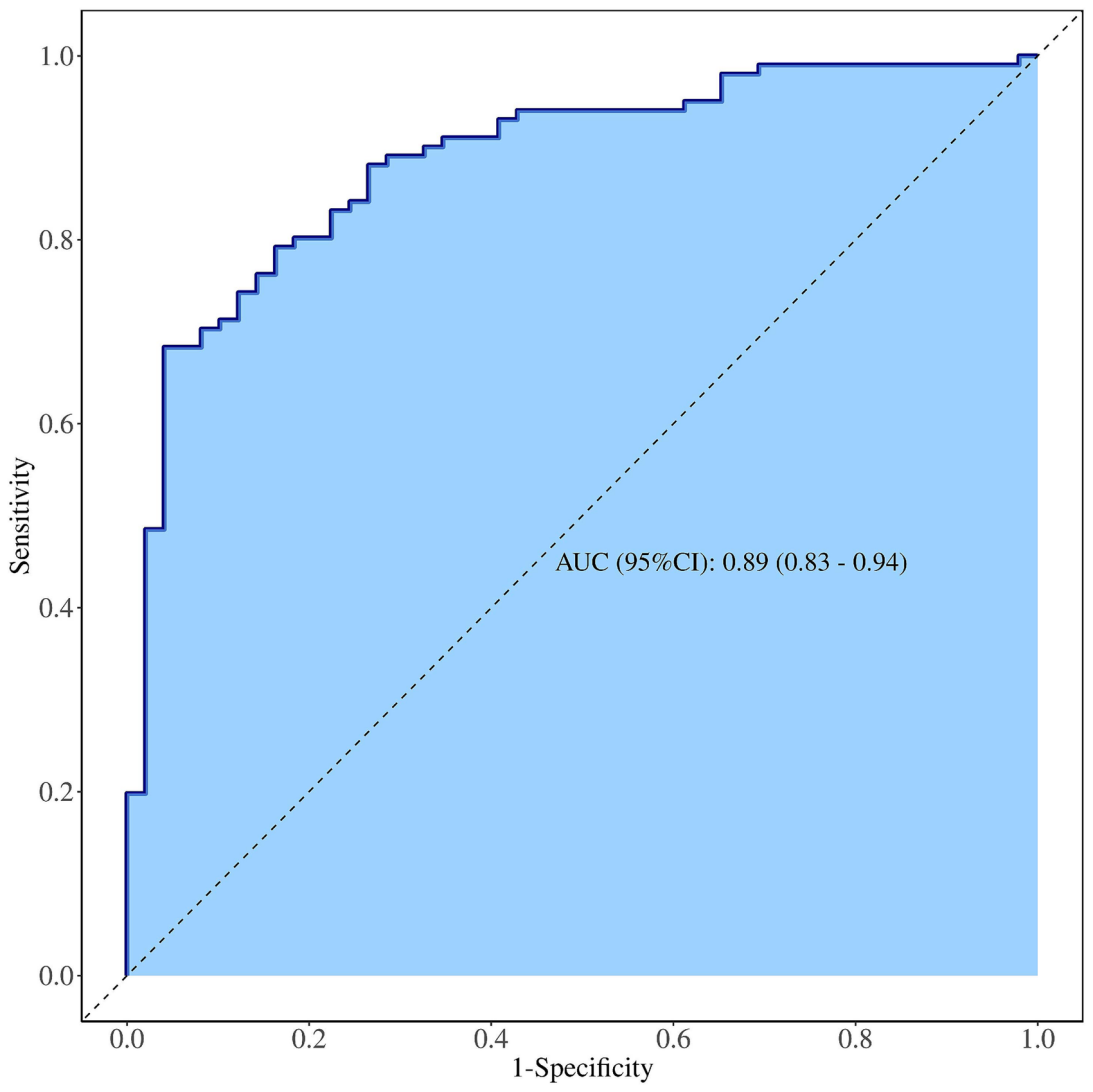
ROC curve of contrast-enhanced ultrasound diagnosis of suspicious breast lesions.

The diagnostic efficacy of the integrated model for suspicious breast lesions is higher than that of independent risk factors (heterogeneous perfusion, crab clam-like enhancement, and partial_ IMAX), with an AUCROC of 0.89 (95% CI: 0.83–0.94). When the cutoff value was 0.608, the sensitivity and specificity were 83.0 and 78.0%, respectively, and the accuracy was 81.3% (Supplementary Table 5 and Figure 4).

In diagnosing benign pathological subtypes of breast lesions, there was no significant difference between independent risk factors and integrated model for diagnosis of FIBs and NPMs (*p* > 0.05). In the diagnosis of malignant pathological subtypes of breast lesions, there was no significant difference between independent risk factors and integrated model in the diagnosis of IDCs and DCISs (*p* > 0.05) (Supplementary Table 5).

## Discussion

In clinical practice, the grayscale ultrasonic images of mass-like NPM and atypical FIB often present risk features similar to those of IDC and DCIS (18, 19), leading to misdiagnoses and subsequent incorrect therapies. Previous reports have shown that CEUS risk features (including enlarged scope, heterogeneous perfusion, perfusion deficiency, crab clam-like enhancement, more than two enhanced vessels within lesions, and surrounding enriched vessels inserting into lesions) are more specific to malignant than benign lesions (10, 20, 21), consistent with our findings in this study (Table 2). However, previous studies did not assess the potential value of these risk features in differentiating various histopathological subtypes of suspicious breast lesions with high US-BI-RADS scores, especially for atypical benign lesions that are easily mistaken for breast cancers. Therefore, in this study, we explored the value of these CEUS risk features in distinguishing IDC, DCIS, mass-like NPM, and atypical FIB categorized as BI-RADS 4.

Our study showed that the qualitative features of enlarged scope, irregular shape, and perfusion deficiency were less frequent in atypical FIB than IDC, DCIS, and NPM, thus enabling the distinction of atypical FIBs from other types of lesions (Figure 2). These three risk features provide physicians with valuable information for differentiating atypical FIB from breast cancers but fail to differentiate NPMs from IDC and DCIS (Figure 2). This may be related to inflammatory responses or bacterial infections occurring in NPMs, which could stimulate vascular proliferation and infiltrating into the surrounding tissue of lesions (22), subsequently leading to risk features such as an enlarged scope and irregular shape. Additionally, the specific feature of surrounding enriched vessels inserting into lesions differentiates NPMs not only from IDC and DCIS but also from atypical FIB (Figure 2). It demonstrated the highest diagnostic sensitivity of 100% and the highest accuracy of 87.3% in distinguishing between benign and malignant breast lesions (Table 2). Thus, among all the risk features of CEUS, surrounding enriched vessels inserting into lesions would be the most specific in differentiating NPM from breast cancers and atypical FIBs.

In contrast, among the quantitative parameters of CEUS, only IMAX contributed to the differential diagnosis between malignant and benign tumors (*p* < 0.05, ROCAUC: 0.61; sensitivity: 60.4%; specificity: 65.9%; accuracy: 62.1%) (Table 3 and Supplementary Figure 2). TomTec SonoLiver software used in this study focuses on the quantitative analysis of liver lesions by CEUS. Unlike diffuse liver disease, breast lesions are often accompanied by calcification and necrotic areas, which appear as heterogeneous perfusion, potentially leading to instability in quantitative curves and parameters and ultimately resulting in low diagnostic efficiency. IMAX did not show any difference in the paired comparison of IDC, DCIS, atypical FIB, and mass-like NPM (Supplementary Table 4 and Supplementary Figure 3). These results indicate that the quantitative parameters of CEUS have limited value in differentiating the histopathological subtypes of suspicious breast lesions, consistent with the previous reports (23, 24).

Although our findings suggest that the quantitative parameters of CEUS have limited value in identifying histopathological subtypes of suspicious breast lesions, the results of integrated model constructed by combining quantitative features and quantitative parameters of CEUS show that the model has high diagnostic efficiency (ROCAUC: 0.89; sensitivity: 83.0%; and accuracy: 78.0%) for identifying category 4 of US-BI-RADS and can better distinguish suspicious breast lesions, consistent with previous reports (Supplementary Table 5) (25–29). Unfortunately, it has limited value in identifying the histopathological subtypes of suspicious breast lesions.

## Conclusion

Some qualitative risk features of CEUS can distinguish malignant breast lesions from NPMs and atypical FIBs with high US-BI-RADS scores, helping physicians reduce the misdiagnosis of suspicious breast lesions in clinical practice.

## Data Availability

The original contributions presented in the study are included in the article/Supplementary material, further inquiries can be directed to the corresponding author.
