# Supplementary material for: The benefits of contrast-enhanced ultrasound in the differential diagnosis of suspicious breast lesions
Source: Front Med (Lausanne). 2024 Dec 24;11:1511200. doi: 10.3389/fmed.2024.1511200 (PMC11703730; doi:10.3389/fmed.2024.1511200)
Supplement: Supplementary file 1 [file SM_Data_Sheet_1511200.docx]

Analysis of quantitative parameters of CEUS for differentitating histopathological subtypes of suspicious breast lesions

#Read the data and show the data structur
setwd("C:/Users/Administrator/Desktop/RESUTLS")
data<-read.csv("quanti_breast_1102.csv")
str(data)

## 'data.frame': 150 obs. of 28 variables:
## $ id : chr "SEQ-056" "SEQ-071" "SEQ-095" "SEQ-097" ...
## $ idn : chr "56" "71" "95" "97" ...
## $ Path1 : chr "纤维腺瘤" "肉芽肿" "肉芽肿" "纤维腺瘤" ...
## $ Path : chr "纤维瘤" "慢性化脓性炎" "肉芽肿性小叶炎伴钙化" "纤维瘤" ...
## $ His : int 0 0 0 0 0 0 0 0 0 0 ...
## $ His_two : int 0 0 0 0 0 0 0 0 0 0 ...
## $ sex : chr "女" "女" "女" "女" ...
## $ age : int 38 30 31 44 51 43 52 41 46 35 ...
## $ norm_IMAX : int 100 100 100 100 100 100 100 100 100 100 ...
## $ norm_RT : num 20.62 8.71 8.89 12.6 11.26 ...
## $ norm_TTP : num 31.1 11.8 12.4 13.2 13.8 ...
## $ norm_mTT : num 119.3 19.4 19.5 105.6 29 ...
## $ norm_QOF : num 78.3 93.3 81.8 59.5 79.6 ...
## $ Whole_IMAX : num 422.8 78.6 257.9 773.8 198.5 ...
## $ Whole_RT : num 9.23 6.9 9.15 5.94 6.44 ...
## $ Whole_TTP : num 12.92 8.46 11.33 7.63 7.04 ...
## $ Whole_mTT : num 20.2 17.8 23.2 14.1 27.9 ...
## $ Whole_QOF : num 98.1 83.8 86.4 98 97.1 ...
## $ Parb_IMAX : num 269.6 39.3 248.1 677.5 324 ...
## $ Parb_RT : num 2.81 7.4 7.32 5.85 5.83 ...
## $ Parb_TTP : num 12.1 10.79 10.1 9.09 8.89 ...
## $ Parb_mTT : num 12.4 15.9 16.1 12.5 12.4 ...
## $ Parb_QOF : num 47.1 79.5 77.8 92.1 75.3 ...
## $ Partial_IMAX: num 627.2 81.9 342.9 967.9 110 ...
## $ Partial_RT : num 7.95 7.22 9.28 5.33 8.02 ...
## $ Partial_TTP : num 12.68 8.43 11.67 8.43 8.56 ...
## $ Partial_mTT : num 17 21.7 22.8 11.4 46.7 ...
## $ Partial_QOF : num 89.5 82.3 85.1 96.1 52 ...

#Analyze the features of basic information from the total patients with suffering the breast lesions.
table(data$Path1,data$His_two)

##
## 0 1
## 导管内癌 0 12
## 导管内乳头状癌 0 2
## 导管内乳头状瘤 4 0
## 浆乳 2 0
## 浸润性癌 0 83
## 肉芽肿 7 0
## 乳腺炎 5 0
## 髓样癌 0 1
## 纤维腺瘤 20 0
## 小叶癌 0 2
## 增生 10 0
## 增生伴纤维腺瘤 1 0
## 粘液癌 0 1

table(data$His_two)

##
## 0 1
## 49 101

table(data$sex)

##
## 男 女
## 1 149

table(data$His_two,data$sex)

##
## 男 女
## 0 1 48
## 1 0 101

mean(data$age)

## [1] 50.09333

sd(data$age)

## [1] 13.21396

cancer<-which(data$His_two==1)
mean(data$age[cancer])

## [1] 52.78218

sd(data$age[cancer])

## [1] 13.46225

benign<-which(data$His_two==0)
mean(data$age[benign])

## [1] 44.55102

sd(data$age[benign])

## [1] 10.85714

t.test(data$age[cancer],data$age[benign])$p.value

## [1] 0.0001055337

#Remove cases with the quality of fit less than 50%
data<-read.csv("quanti_breast_1102.csv")
cut_Anal<-which(data$Whole_QOF<50)
cut_Parm<-which(data$Partial_QOF<50)
cut<-union(cut_Anal,cut_Parm)
data.out<-data[cut,]
table(data.out$Path1,data.out$His_two)

##
## 0 1
## 浸润性癌 0 3
## 肉芽肿 1 0
## 纤维腺瘤 1 0
## 小叶癌 0 1
## 增生 2 0
## 增生伴纤维腺瘤 1 0
## 粘液癌 0 1

data<-data[-cut,]

#The mean and standard deviation of CEUS parameters
benig_data<-data[which(data$His_two==0),9:28]
cancer_data<-data[which(data$His_two==1),9:28]
benig_mean<-apply(benig_data,2,mean)
cancer_mean<-apply(cancer_data,2,mean)
benig_sd<-apply(benig_data,2,sd)
cancer_sd<-apply(cancer_data,2,sd)
cbind(benig_mean,benig_sd)

## benig_mean benig_sd
## norm_IMAX 100.000000 0.000000
## norm_RT 11.076591 7.191759
## norm_TTP 16.594773 13.040248
## norm_mTT 45.560455 52.859074
## norm_QOF 75.016591 11.843921
## Whole_IMAX 226.785909 210.415191
## Whole_RT 10.053182 4.877301
## Whole_TTP 12.432500 5.894636
## Whole_mTT 38.622045 51.866173
## Whole_QOF 83.713409 12.756657
## Parb_IMAX 275.291818 222.472585
## Parb_RT 10.654773 8.482136
## Parb_TTP 14.569091 10.014768
## Parb_mTT 36.686364 52.430972
## Parb_QOF 75.653409 14.349059
## Partial_IMAX 329.647045 325.550237
## Partial_RT 8.346818 4.928044
## Partial_TTP 12.941364 8.099005
## Partial_mTT 27.151364 23.598915
## Partial_QOF 80.062727 11.433652

cbind(cancer_mean,cancer_sd)

## cancer_mean cancer_sd
## norm_IMAX 100.000000 0.000000
## norm_RT 10.240104 5.584902
## norm_TTP 16.343958 7.628535
## norm_mTT 33.485833 35.526353
## norm_QOF 77.987500 9.315086
## Whole_IMAX 411.250417 482.857184
## Whole_RT 8.838125 3.156522
## Whole_TTP 11.471875 4.169958
## Whole_mTT 26.315833 18.279235
## Whole_QOF 85.993229 13.210341
## Parb_IMAX 507.736979 648.325616
## Parb_RT 8.219375 4.358852
## Parb_TTP 12.429688 5.834209
## Parb_mTT 25.112604 19.985500
## Parb_QOF 79.887396 13.964490
## Partial_IMAX 629.027812 1074.104048
## Partial_RT 7.520833 3.396983
## Partial_TTP 11.748125 5.017862
## Partial_mTT 22.368542 25.675625
## Partial_QOF 82.772396 9.523456

#p values of quantitative parameters to differentiate the benign from malignant breast lesions
pp<-function(x,y){
 pvalues<-rep(0,20)
 pvalues[1]<-1
 for(i in 2:20){
 ttmp<-t.test(x[,i],y[,i])$p.value
 pvalues[i]<-ttmp
}
return(pvalues)
}

pp(benig_data,cancer_data)

## [1] 1.000000000 0.497000578 0.905994972 0.172838497 0.146512657 0.002019287
## [7] 0.135370337 0.333285835 0.132366140 0.334422259 0.002132835 0.077674060
## [13] 0.192725546 0.162602241 0.106028682 0.013983318 0.317590111 0.371158803
## [19] 0.281944902 0.175191503

#Draw the boxplot of quantitative parameters of CEUS in the three masked interest of regions, including the normal breast gland tissue, the whole and the part of the enhanced lesions.
data<-read.csv("quanti_breast_1102.csv")
cut_Whole<-which(data$Whole_QOF<50)
cut_Partial<-which(data$Partial_QOF<50)
cut<-union(cut_Whole,cut_Partial)
data.out<-data[cut,]
table(data.out$Path1,data.out$His_two)

##
## 0 1
## 浸润性癌 0 3
## 肉芽肿 1 0
## 纤维腺瘤 1 0
## 小叶癌 0 1
## 增生 2 0
## 增生伴纤维腺瘤 1 0
## 粘液癌 0 1

data<-data[-cut,]

par(mar=c(3,4,1,1))
par(mfrow=c(1,1))
data$His_two[which(data$His_two==0)]<-rep("Benignity",length(which(data$His_two==0)))
data$His_two[which(data$His_two==1)]<-rep("Malignancy",length(which(data$His_two==1)))

#the quality of fit
QOF_data<-cbind(c(data$norm_QOF,data$Whole_QOF,data$Partial_QOF),c(rep("Normal",dim(data)[1]),rep("Whole",dim(data)[1]),rep("Partial",dim(data)[1])))
QOF<-as.data.frame(QOF_data)
colnames(QOF)<-c("qof","pos")
QOF$qof<-as.numeric(QOF$qof)
boxplot(qof ~ pos,data=QOF,lwd=1,xlab="",ylab="",font=4,boxwex=0.25)
mtext("Quality of fit (QOF)",side=2,font=1,cex=1.2,line=2.5)


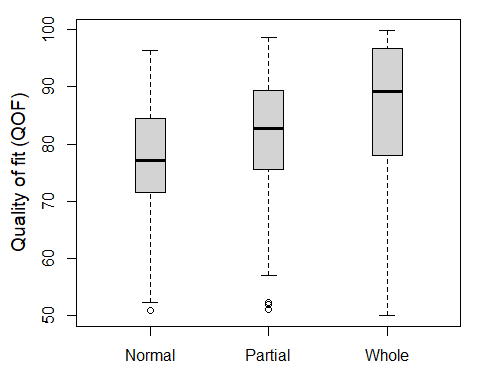


#the maximum intensity
ben<-which(data$His_two=="Benignity")
IMAX_ben<-cbind(c(data$norm_IMAX[ben],data$Whole_IMAX[ben],data$Partial_IMAX[ben]),c(rep("Normal",length(ben)),rep("Whole",length(ben)),rep("Partial",length(ben))))
IMAX_ben<-as.data.frame(IMAX_ben)
colnames(IMAX_ben)<-c("max","pos")
IMAX_ben$max<-as.numeric(IMAX_ben$max)

mag<-which(data$His_two=="Malignancy")
IMAX_mag<-cbind(c(data$norm_IMAX[mag],data$Whole_IMAX[mag],data$Partial_IMAX[mag]),c(rep("Normal",length(mag)),rep("Whole",length(mag)),rep("Partial",length(mag))))
IMAX_mag<-as.data.frame(IMAX_mag)
colnames(IMAX_mag)<-c("max","pos")
IMAX_mag$max<-as.numeric(IMAX_mag$max)

par(mar=c(3,4,1,1))
par(mfrow=c(1,1))
#remove the two outvalues
data$Path1[which(data$Partial_IMAX>4000)]

## [1] "浸润性癌" "浸润性癌"

outvalue1<-which(IMAX_mag[,1]>4000)
IMAX_mag<-IMAX_mag[-outvalue1,]
boxplot(max ~ pos,data=IMAX_ben,lwd=1,xlab="",ylab="",font=4,col=2,boxwex=0.25,ylim=c(0,3000),pch=16)
boxplot(max ~ pos,data=IMAX_mag,lwd=1,xlab="",ylab="",font=4,col=3,add=TRUE,at=1:3+0.3,boxwex=0.25,names=FALSE,pch=16)
mtext("Maximum Intensity (IMAX)",side=2,font=1,cex=1.2,line=2.5)
nam<-c("Benignant breast lesion","Malignant breast lesion")
legend("topright",nam,pch=15,bty="n",col=2:3,cex=1,text.col=2:3,text.font=4)


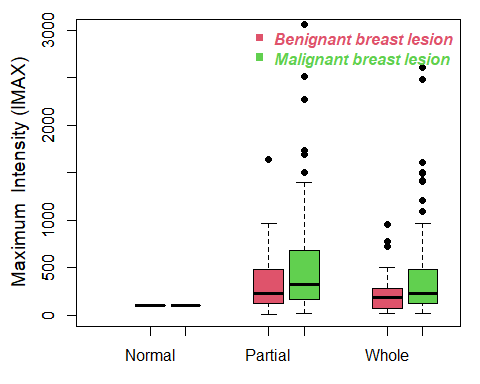


#the rise time
ben<-which(data$His_two=="Benignity")
RT_ben<-cbind(c(data$norm_RT[ben],data$Whole_RT[ben],data$Partial_RT[ben]),c(rep("Normal",length(ben)),rep("Whole",length(ben)),rep("Partial",length(ben))))
RT_ben<-as.data.frame(RT_ben)
colnames(RT_ben)<-c("rt","pos")
RT_ben$rt<-as.numeric(RT_ben$rt)

mag<-which(data$His_two=="Malignancy")
RT_mag<-cbind(c(data$norm_RT[mag],data$Whole_RT[mag],data$Partial_RT[mag]),c(rep("Normal",length(mag)),rep("Whole",length(mag)),rep("Partial",length(mag))))
RT_mag<-as.data.frame(RT_mag)
colnames(RT_mag)<-c("rt","pos")
RT_mag$rt<-as.numeric(RT_mag$rt)

par(mar=c(3,4,1,1))
par(mfrow=c(1,1))
boxplot(rt ~ pos,data=RT_ben,lwd=1,xlab="",ylab="",font=4,col=2,boxwex=0.25,pch=16)
boxplot(rt ~ pos,data=RT_mag,lwd=1,xlab="",ylab="",font=4,col=3,add=TRUE,at=1:3+0.3,boxwex=0.25,names=FALSE,pch=16)
mtext("Rise Time (RT)",side=2,font=1,cex=1.2,line=2.5)
nam<-c("Benignant breast lesion","Malignant breast lesion")
legend("topright",nam, pch = 15,bty="n",col=2:3,cex=1,text.col=2:3,text.font=4)


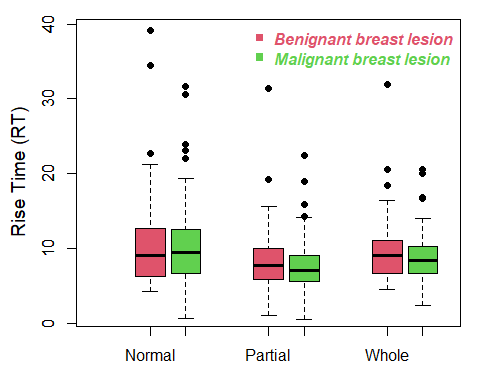


#the time to peak
ben<-which(data$His_two=="Benignity")
TTP_ben<-cbind(c(data$norm_TTP[ben],data$Whole_TTP[ben],data$Partial_TTP[ben]),c(rep("Normal",length(ben)),rep("Whole",length(ben)),rep("Partial",length(ben))))
TTP_ben<-as.data.frame(TTP_ben)
colnames(TTP_ben)<-c("ttp","pos")
TTP_ben$ttp<-as.numeric(TTP_ben$ttp)

mag<-which(data$His_two=="Malignancy")
TTP_mag<-cbind(c(data$norm_TTP[mag],data$Whole_TTP[mag],data$Partial_TTP[mag]),c(rep("Normal",length(mag)),rep("Whole",length(mag)),rep("Partial",length(mag))))
TTP_mag<-as.data.frame(TTP_mag)
colnames(TTP_mag)<-c("ttp","pos")
TTP_mag$ttp<-as.numeric(TTP_mag$ttp)

par(mar=c(3,4,1,1))
par(mfrow=c(1,1))
#remove the outvalues
outvalue3<-which(TTP_ben[,1]>60)
TTP_ben<-TTP_ben[-outvalue3,]
boxplot(ttp ~ pos,data=TTP_ben,lwd=1,xlab="",ylab="",font=4,col=2,boxwex=0.25,pch=16)
boxplot(ttp ~ pos,data=TTP_mag,lwd=1,xlab="",ylab="",font=4,col=3,add=TRUE,at=1:3+0.3,boxwex=0.25,names=FALSE,pch=16)
mtext("Time to peak (TTP)",side=2,font=1,cex=1.2,line=2.5)
nam<-c("Benignant breast lesion","Malignant breast lesion")
legend("topright",nam, pch = 15,bty="n",col=2:3,cex=1,text.col=2:3,text.font=4)


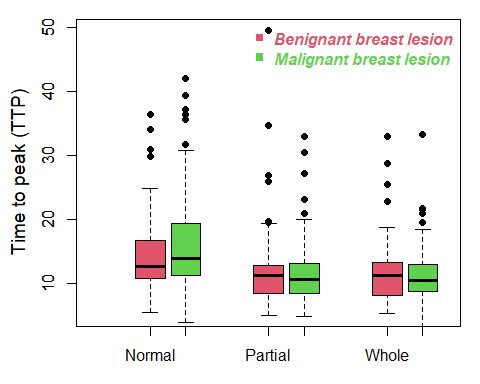


#the mean transit time
ben<-which(data$His_two=="Benignity")
mTT_ben<-cbind(c(data$norm_mTT[ben],data$Whole_mTT[ben],data$Partial_mTT[ben]),c(rep("Normal",length(ben)),rep("Whole",length(ben)),rep("Partial",length(ben))))
mTT_ben<-as.data.frame(mTT_ben)
colnames(mTT_ben)<-c("mTT","pos")
mTT_ben$mTT<-as.numeric(mTT_ben$mTT)

mag<-which(data$His_two=="Malignancy")
mTT_mag<-cbind(c(data$norm_mTT[mag],data$Whole_mTT[mag],data$Partial_mTT[mag]),c(rep("Normal",length(mag)),rep("Whole",length(mag)),rep("Partial",length(mag))))
mTT_mag<-as.data.frame(mTT_mag)
colnames(mTT_mag)<-c("mTT","pos")
mTT_mag$mTT<-as.numeric(mTT_mag$mTT)

par(mar=c(3,4,1,1))
par(mfrow=c(1,1))
#remove outvalue
outvalue4<-which(mTT_ben[,1]>200)
mTT_ben<-mTT_ben[-outvalue4,]
outvalue5<-which(mTT_mag[,1]>200)
mTT_mag<-mTT_mag[-outvalue5,]
boxplot(mTT ~ pos,data=mTT_ben,lwd=1,xlab="",ylab="",font=4,col=2,boxwex=0.25,ylim=c(0,200),pch=16)
boxplot(mTT ~ pos,data=mTT_mag,lwd=1,xlab="",ylab="",font=4,col=3,add=TRUE,at=1:3+0.3,boxwex=0.25,names=FALSE,pch=16)
mtext("Mean Transit Time (mTT)",side=2,font=1,cex=1.2,line=2.5)
nam<-c("Benignant breast lesion","Malignant breast lesion")
legend("topright",nam, pch = 15,bty="n",col=2:3,cex=1,text.col=2:3,text.font=4)


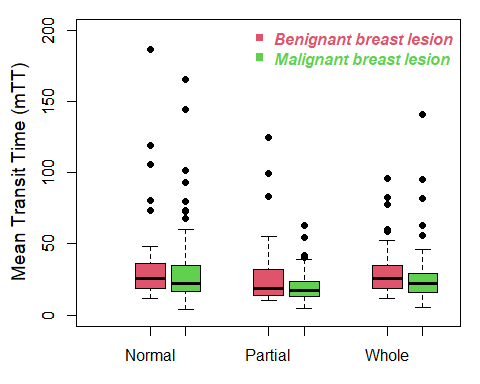


#Comparison of ROC curves of four quantitative parameters of CEUS within the whole lesion masked
#IMAX
library(pROC)
Whole_IMAX<-roc(data$His_two, data$Whole_IMAX,ci=TRUE)
tmp.roc<-cbind(Whole_IMAX$sensitivities,Whole_IMAX$specificities,Whole_IMAX$thresholds)
#sensitivity, specificity and thresholds
tmp.roc[which.max(tmp.roc[,1]+tmp.roc[,2]),]

## [1] 0.4479167 0.7727273 299.4200000

#the area under the ROC curve
Whole_IMAX$auc

## Area under the curve: 0.6171

#confidence interval
Whole_IMAX$ci

## 95% CI: 0.5195-0.7146 (DeLong)

scores<- which(data$Whole_IMAX>tmp.roc[which.max(tmp.roc[,1]+tmp.roc[,2]),][3])
data$Whole_IMAX[scores]<-rep(1,length(scores))
data$Whole_IMAX[-scores]<-rep(0,dim(data)[1]-length(scores))
tab_count<-table(data$His_two,data$Whole_IMAX)
tab_count

##
## 0 1
## Benignity 34 10
## Malignancy 53 43

#p value of chi-square test
chisq.test(tab_count)$p.value

## [1] 0.02082938

#the diagnostic accuracy
ACC<-(tab_count[1,1]+tab_count[2,2])/sum(tab_count)
ACC

## [1] 0.55

#Negative predictive test
NPV<-(tab_count[1,1])/colSums(tab_count)[1]
NPV

## 0
## 0.3908046

#Positive predictive test
PPV<-(tab_count[2,2])/colSums(tab_count)[2]
PPV

## 1
## 0.8113208

#ROC curve of RT
Whole_RT<-roc(data$His_two, data$Whole_RT ,ci=TRUE)
tmp.roc<-cbind(Whole_RT$sensitivities,Whole_RT$specificities,Whole_RT$thresholds)
#sensitivity, specificity and thresholds
tmp.roc[which.max(tmp.roc[,1]+tmp.roc[,2]),]

## [1] 0.5937500 0.5454545 8.9750000

#the area under the ROC curve
Whole_RT$auc

## Area under the curve: 0.5607

#confidence interval
Whole_RT$ci

## 95% CI: 0.4552-0.6663 (DeLong)

scores<- which(data$Whole_RT>tmp.roc[which.max(tmp.roc[,1]+tmp.roc[,2]),][3])
data$Whole_RT[scores]<-rep(0,length(scores))
data$Whole_RT[-scores]<-rep(1,dim(data)[1]-length(scores))
tab_count<-table(data$His_two,data$Whole_RT)
tab_count

##
## 0 1
## Benignity 24 20
## Malignancy 39 57

#p value of chi-square test
chisq.test(tab_count)$p.value

## [1] 0.1757388

#the diagnostic accuracy
ACC<-(tab_count[1,1]+tab_count[2,2])/sum(tab_count)
ACC

## [1] 0.5785714

#Negative predictive test
NPV<-(tab_count[1,1])/colSums(tab_count)[1]
NPV

## 0
## 0.3809524

#Positive predictive test
PPV<-(tab_count[2,2])/colSums(tab_count)[2]
PPV

## 1
## 0.7402597

#ROC curve of TTP
Whole_TTP<-roc(data$His_two, data$Whole_TTP ,ci=TRUE)
tmp.roc<-cbind(Whole_TTP$sensitivities,Whole_TTP$specificities,Whole_TTP$thresholds)
#sensitivity, specificity and thresholds
tmp.roc[which.max(tmp.roc[,1]+tmp.roc[,2]),]

## [1] 0.9270833 0.1818182 16.9000000

#the area under the ROC curve
Whole_TTP$auc

## Area under the curve: 0.5141

#confidence interval
Whole_TTP$ci

## 95% CI: 0.405-0.6231 (DeLong)

scores<- which(data$Whole_TTP>tmp.roc[which.max(tmp.roc[,1]+tmp.roc[,2]),][3])
data$Whole_TTP[scores]<-rep(0,length(scores))
data$Whole_TTP[-scores]<-rep(1,dim(data)[1]-length(scores))
tab_count<-table(data$His_two,data$Whole_TTP)
tab_count

##
## 0 1
## Benignity 8 36
## Malignancy 7 89

#p value of chi-square test
chisq.test(tab_count)$p.value

## [1] 0.1010663

#the diagnostic accuracy
ACC<-(tab_count[1,1]+tab_count[2,2])/sum(tab_count)
ACC

## [1] 0.6928571

#the negative predictive test
NPV<-(tab_count[1,1])/colSums(tab_count)[1]
NPV

## 0
## 0.5333333

#the positive predictive test
PPV<-(tab_count[2,2])/colSums(tab_count)[2]
PPV

## 1
## 0.712

#ROC curve of mTT
Whole_mTT<-roc(data$His_two, data$Whole_mTT ,ci=TRUE)
tmp.roc<-cbind(Whole_mTT$sensitivities,Whole_mTT$specificities,Whole_mTT$thresholds)
#sensitivity, specificity and cutoff
tmp.roc[which.max(tmp.roc[,1]+tmp.roc[,2]),]

## [1] 0.7604167 0.4545455 29.6350000

#the area under the ROC curve
Whole_mTT$auc

## Area under the curve: 0.605

#confidence interval
Whole_mTT$ci

## 95% CI: 0.5018-0.7082 (DeLong)

scores<- which(data$Whole_mTT>tmp.roc[which.max(tmp.roc[,1]+tmp.roc[,2]),][3])
data$Whole_mTT[scores]<-rep(0,length(scores))
data$Whole_mTT[-scores]<-rep(1,dim(data)[1]-length(scores))
tab_count<-table(data$His_two,data$Whole_mTT)
tab_count

##
## 0 1
## Benignity 20 24
## Malignancy 23 73

#p value of chi-square test
chisq.test(tab_count)$p.value

## [1] 0.01816435

#the diagnostic accuracy
ACC<-(tab_count[1,1]+tab_count[2,2])/sum(tab_count)
ACC

## [1] 0.6642857

#the negative predictive value
NPV<-(tab_count[1,1])/colSums(tab_count)[1]
NPV

## 0
## 0.4651163

#the positive predictive value
PPV<-(tab_count[2,2])/colSums(tab_count)[2]
PPV

## 1
## 0.7525773

#Draw the ROCs of the four quantitative parameters of CEUS within the whole lesion masked
data<-read.csv("quanti_breast_1102.csv")
cut_Whole<-which(data$Whole_QOF<50)
cut_Partial<-which(data$Partial_QOF<50)
cut<-union(cut_Whole,cut_Partial)
data<-data[-cut,]
par(mar=c(5,5,1,1))
par(mfrow=c(1,1))
Whole_IMAX<-roc(data$His_two, data$Whole_IMAX,ci=TRUE)
Whole_RT<-roc(data$His_two, data$Whole_RT ,ci=TRUE)
Whole_TTP<-roc(data$His_two, data$Whole_TTP ,ci=TRUE)
Whole_mTT<-roc(data$His_two, data$Whole_mTT ,ci=TRUE)

colrs<-c("black","red","green","blue","cyan",colors()[548],colors()[500],colors()[567])
plot(1-Whole_IMAX$specificities,Whole_IMAX$sensitivities,type="l",lwd=3,xlab="",ylab="",xaxt="n",yaxt="n",col=colrs[1])
axis(1,seq(0,1,by=0.1),cex.axis=1.3,font=4,las=1.5)
axis(2,cex.axis=1.3,las=2,font=4)
mtext("1-Specificity",side=1,font=4,cex=1.3,line=3)
mtext("Sensitivity",side=2,font=4,cex=1.3,line=3.3)
points(1-Whole_RT$specificities,Whole_RT$sensitivities,type="l",lwd=3,xlab="",ylab="",xaxt="n",yaxt="n",col=colrs[2])
points(1-Whole_TTP$specificities,Whole_TTP$sensitivities,type="l",lwd=3,xlab="",ylab="",xaxt="n",yaxt="n",col=colrs[3])
points(1-Whole_mTT$specificities,Whole_mTT$sensitivities,type="l",lwd=3,xlab="",ylab="",xaxt="n",yaxt="n",col=colrs[4])
abline(a=0,b=1,col="gray80",lwd=3,lty=2)
nam<-c("Whole_IMAX(AUC=0.62)","Whole_RT(AUC=0.56)","Whole_TTP(AUC=0.51)","Whole_mTT(AUC=0.61)")
legend("bottomright",nam, pch = 15,bty="n",col=colrs,cex=1,text.col=colrs[1:4],text.font=4)


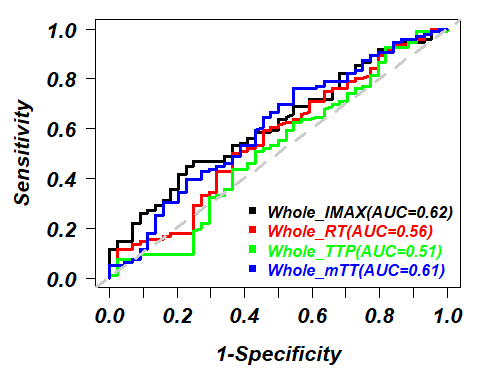


#there is no difference between IMAX and mTT in the whole lesion
roc.test(Whole_IMAX,Whole_mTT)$p.value

## [1] 0.8283207

#Draw the ROCs of the four quantitative parameters of CEUS within the part of lesion with solid composition masked
data<-read.csv("quanti_breast_1102.csv")
cut_Whole<-which(data$Whole_QOF<50)
cut_Partial<-which(data$Partial_QOF<50)
cut<-union(cut_Whole,cut_Partial)
data<-data[-cut,]
library(pROC)
Partial_IMAX<-roc(data$His_two, data$Partial_IMAX,ci=TRUE)
tmp.roc<-cbind(Partial_IMAX$sensitivities,Partial_IMAX$specificities,Partial_IMAX$thresholds)
#sensitivity, specificity and thresholds
tmp.roc[which.max(tmp.roc[,1]+tmp.roc[,2]),]

## [1] 0.6041667 0.6590909 267.3450000

#the area under the ROC curve
Partial_IMAX$auc

## Area under the curve: 0.6077

#confidence interval
Partial_IMAX$ci

## 95% CI: 0.5083-0.7071 (DeLong)

scores<- which(data$Partial_IMAX>tmp.roc[which.max(tmp.roc[,1]+tmp.roc[,2]),][3])
data$Partial_IMAX[scores]<-rep(1,length(scores))
data$Partial_IMAX[-scores]<-rep(0,dim(data)[1]-length(scores))
tab_count<-table(data$His_two,data$Partial_IMAX)
tab_count

##
## 0 1
## 0 29 15
## 1 38 58

#the diagnostic accuracy
(tab_count[1,1]+tab_count[2,2])/sum(tab_count)

## [1] 0.6214286

#p value of chi-square test
chisq.test(tab_count)$p.value

## [1] 0.006677627

#the diagnostic accuracy
ACC<-(tab_count[1,1]+tab_count[2,2])/sum(tab_count)
ACC

## [1] 0.6214286

#Negative predictive test
NPV<-(tab_count[1,1])/colSums(tab_count)[1]
NPV

## 0
## 0.4328358

#Positive predictive test
PPV<-(tab_count[2,2])/colSums(tab_count)[2]
PPV

## 1
## 0.7945205

###ROC curve of RT
Partial_RT<-roc(data$His_two, data$Partial_RT ,ci=TRUE)
tmp.roc<-cbind(Partial_RT$sensitivities,Partial_RT$specificities,Partial_RT$thresholds)
#sensitivity, specificity and thresholds
tmp.roc[which.max(tmp.roc[,1]+tmp.roc[,2]),]

## [1] 0.5729167 0.5227273 7.6700000

#the area under the ROC curve
Partial_RT$auc

## Area under the curve: 0.5398

#confidence interval
Partial_RT$ci

## 95% CI: 0.4349-0.6446 (DeLong)

scores<- which(data$Partial_RT>tmp.roc[which.max(tmp.roc[,1]+tmp.roc[,2]),][3])
data$Partial_RT[scores]<-rep(0,length(scores))
data$Partial_RT[-scores]<-rep(1,dim(data)[1]-length(scores))
tab_count<-table(data$His_two,data$Partial_RT)
tab_count

##
## 0 1
## 0 23 21
## 1 41 55

#p value of chi-square test
chisq.test(tab_count)$p.value

## [1] 0.3832793

#the diagnostic accuracy
ACC<-(tab_count[1,1]+tab_count[2,2])/sum(tab_count)
ACC

## [1] 0.5571429

#Negative predictive test
NPV<-(tab_count[1,1])/colSums(tab_count)[1]
NPV

## 0
## 0.359375

#Positive predictive test
PPV<-(tab_count[2,2])/colSums(tab_count)[2]
PPV

## 1
## 0.7236842

###ROC curve of TTP
Partial_TTP<-roc(data$His_two, data$Partial_TTP ,ci=TRUE)
tmp.roc<-cbind(Partial_TTP$sensitivities,Partial_TTP$specificities,Partial_TTP$thresholds)
#sensitivity, specificity and thresholds
tmp.roc[which.max(tmp.roc[,1]+tmp.roc[,2]),]

## [1] 0.5416667 0.5909091 10.7050000

#the area under the ROC curve
Partial_TTP$auc

## Area under the curve: 0.5212

#confidence interval
Partial_TTP$ci

## 95% CI: 0.4154-0.627 (DeLong)

scores<- which(data$Partial_TTP>tmp.roc[which.max(tmp.roc[,1]+tmp.roc[,2]),][3])
data$Partial_TTP[scores]<-rep(0,length(scores))
data$Partial_TTP[-scores]<-rep(1,dim(data)[1]-length(scores))
tab_count<-table(data$His_two,data$Partial_TTP)
tab_count

##
## 0 1
## 0 26 18
## 1 44 52

#p value of chi-square test
chisq.test(tab_count)$p.value

## [1] 0.2025276

#the diagnostic accuracy
ACC<-(tab_count[1,1]+tab_count[2,2])/sum(tab_count)
ACC

## [1] 0.5571429

#Negative predictive test
NPV<-(tab_count[1,1])/colSums(tab_count)[1]
NPV

## 0
## 0.3714286

#Positive predictive test
PPV<-(tab_count[2,2])/colSums(tab_count)[2]
PPV

## 1
## 0.7428571

#ROC curve of mTT
Partial_mTT<-roc(data$His_two, data$Partial_mTT ,ci=TRUE)
tmp.roc<-cbind(Partial_mTT$sensitivities,Partial_mTT$specificities,Partial_mTT$thresholds)
#sensitivity, specificity and thresholds
tmp.roc[which.max(tmp.roc[,1]+tmp.roc[,2]),]

## [1] 0.8854167 0.2727273 31.2250000

#the area under the ROC curve
Partial_mTT$auc

## Area under the curve: 0.5568

#confidence interval
Partial_mTT$ci

## 95% CI: 0.4496-0.664 (DeLong)

scores<- which(data$Partial_mTT>tmp.roc[which.max(tmp.roc[,1]+tmp.roc[,2]),][3])
data$Partial_mTT[scores]<-rep(0,length(scores))
data$Partial_mTT[-scores]<-rep(1,dim(data)[1]-length(scores))
tab_count<-table(data$His_two,data$Partial_mTT)
tab_count

##
## 0 1
## 0 12 32
## 1 11 85

#p value of chi-square test
chisq.test(tab_count)$p.value

## [1] 0.03584504

#the diagnostic accuracy
ACC<-(tab_count[1,1]+tab_count[2,2])/sum(tab_count)
ACC

## [1] 0.6928571

#Negative predictive test
NPV<-(tab_count[1,1])/colSums(tab_count)[1]
NPV

## 0
## 0.5217391

#Positive predictive test
PPV<-(tab_count[2,2])/colSums(tab_count)[2]
PPV

## 1
## 0.7264957

#there is no difference between IMAX and mTT in the whole lesion
roc.test(Partial_IMAX,Partial_mTT)$p.value

## [1] 0.4109668

#draw the ROCs of the four quantitative parameters on the partial lesion
data<-read.csv("quanti_breast_1102.csv")
cut_Whole<-which(data$Whole_QOF<50)
cut_Partial<-which(data$Partial_QOF<50)
cut<-union(cut_Anal,cut_Partial)
data<-data[-cut,]
par(mar=c(5,5,1,1))
par(mfrow=c(1,1))
Partial_IMAX<-roc(data$His_two, data$Partial_IMAX,ci=TRUE)
Partial_RT<-roc(data$His_two, data$Partial_RT ,ci=TRUE)
Partial_TTP<-roc(data$His_two, data$Partial_TTP ,ci=TRUE)
Partial_mTT<-roc(data$His_two, data$Partial_mTT ,ci=TRUE)
colrs<-c("black","red","green","blue","cyan",colors()[548],colors()[500],colors()[567])
plot(1-Partial_IMAX$specificities,Partial_IMAX$sensitivities,type="l",lwd=3,xlab="",ylab="",xaxt="n",yaxt="n",col=colrs[1])
axis(1,seq(0,1,by=0.1),cex.axis=1.3,font=4,las=1.5)
axis(2,cex.axis=1.3,las=2,font=4)
mtext("1-Specificity",side=1,font=4,cex=1.3,line=3)
mtext("Sensitivity",side=2,font=4,cex=1.3,line=3.3)
points(1-Partial_RT$specificities,Partial_RT$sensitivities,type="l",lwd=3,xlab="",ylab="",xaxt="n",yaxt="n",col=colrs[2])
points(1-Partial_TTP$specificities,Partial_TTP$sensitivities,type="l",lwd=3,xlab="",ylab="",xaxt="n",yaxt="n",col=colrs[3])
points(1-Partial_mTT$specificities,Partial_mTT$sensitivities,type="l",lwd=3,xlab="",ylab="",xaxt="n",yaxt="n",col=colrs[4])
abline(a=0,b=1,col="gray80",lwd=3,lty=2)
nam<-c("Partial_IMAX(AUC=0.61)","Partial_RT(AUC=0.54)","Partial_TTP(AUC=0.52)","Partial_mTT(AUC=0.56)")
legend("bottomright",nam, pch = 15,bty="n",col=colrs,cex=1,text.col=colrs[1:4],text.font=4)


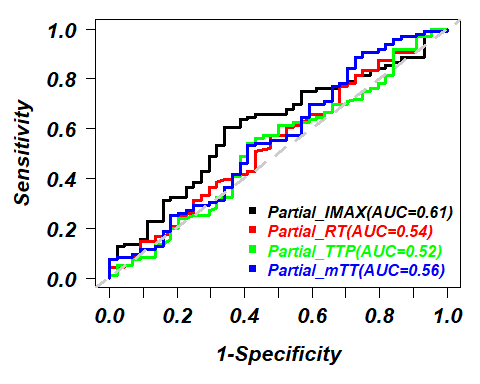


#Comparison of ROCs of IMAX based on the three ROIs of the enhanced lesions
data<-read.csv("quanti_breast_1102.csv")
cut_Whole<-which(data$Whole_QOF<50)
cut_Partial<-which(data$Partial_QOF<50)
cut<-union(cut_Anal,cut_Partial)
data<-data[-cut,]
par(mar=c(5,5,1,1))
par(mfrow=c(1,1))
norm_IMAX<-roc(data$His_two, data$norm_IMAX,ci=TRUE)
Whole_IMAX<-roc(data$His_two, data$Whole_IMAX,ci=TRUE)
Partial_IMAX<-roc(data$His_two, data$Partial_IMAX,ci=TRUE)
colrs<-c("black","red","green","blue","cyan",colors()[548],colors()[500],colors()[567])
plot(1-norm_IMAX$specificities,norm_IMAX$sensitivities,type="l",lwd=3,xlab="",ylab="",xaxt="n",yaxt="n",col=colrs[1],pch=15)
axis(1,seq(0,1,by=0.1),cex.axis=1.3,font=4,las=1.5)
axis(2,cex.axis=1.3,las=2,font=4)
mtext("1-Specificity",side=1,font=4,cex=1.5,line=3)
mtext("Sensitivity",side=2,font=4,cex=1.5,line=3.3)
points(1-Whole_IMAX$specificities,Whole_IMAX$sensitivities,type="l",lwd=3,xlab="",ylab="",xaxt="n",yaxt="n",col=colrs[5],pch=15)
points(1-Partial_IMAX$specificities,Partial_IMAX$sensitivities,type="l",lwd=3,xlab="",ylab="",xaxt="n",yaxt="n",col=colrs[6],pch=15)
abline(a=0,b=1,col="gray80",lwd=3,lty=2)
nam<-c("Norm_IMAX(AUC=0.5)","Whole_IMAX(AUC=0.62)","Partial_IMAX(AUC=0.61)")
legend("bottomright",nam, pch = 15,bty="n",col=colrs,cex=1,text.col=colrs[c(1,5:6)],text.font=4)


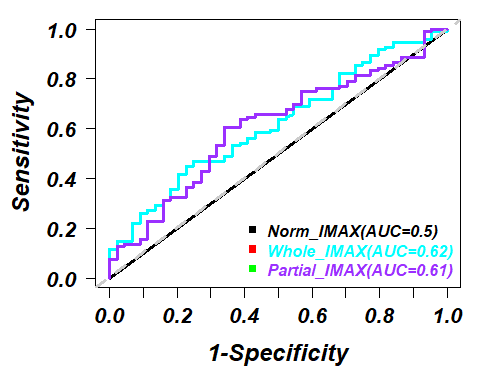


#the test amongst of ref_IMAX, Anal_IMAX and Parm_IMAX
roc.test(Whole_IMAX,Partial_IMAX)$p.value

## [1] 0.7047529

roc.test(Whole_IMAX,norm_IMAX)$p.value

## [1] 0.01869967

roc.test(Partial_IMAX,norm_IMAX)$p.value

## [1] 0.03365158

#Comparison of ROCs of mTT based on the three ROIs of the enhanced lesions
data<-read.csv("quanti_breast_1102.csv")
cut_Whole<-which(data$Whole_QOF<50)
cut_Partial<-which(data$Partial_QOF<50)
cut<-union(cut_Whole,cut_Partial)
data<-data[-cut,]
par(mar=c(5,5,1,1))
par(mfrow=c(1,1))
norm_mTT<-roc(data$His_two, data$norm_mTT,ci=TRUE)
norm_mTT$auc

## Area under the curve: 0.5644

Whole_mTT<-roc(data$His_two, data$Whole_mTT,ci=TRUE)
Whole_mTT$auc

## Area under the curve: 0.605

Partial_mTT<-roc(data$His_two, data$Partial_mTT,ci=TRUE)
Partial_mTT$auc

## Area under the curve: 0.5568

colrs<-c("black","red","green","blue","cyan",colors()[548],colors()[500],colors()[567])
plot(1-norm_mTT$specificities,norm_mTT$sensitivities,type="l",lwd=3,xlab="",ylab="",xaxt="n",yaxt="n",col=colrs[1],pch=15)
axis(1,seq(0,1,by=0.1),cex.axis=1.3,font=4,las=1.5)
axis(2,cex.axis=1.3,las=2,font=4)
mtext("1-Specificity",side=1,font=4,cex=1.5,line=3)
mtext("Sensitivity",side=2,font=4,cex=1.5,line=3.3)
points(1-Whole_mTT$specificities,Whole_mTT$sensitivities,type="l",lwd=3,xlab="",ylab="",xaxt="n",yaxt="n",col=colrs[5],pch=15)
points(1-Partial_mTT$specificities,Partial_mTT$sensitivities,type="l",lwd=3,xlab="",ylab="",xaxt="n",yaxt="n",col=colrs[6],pch=15)
abline(a=0,b=1,col="gray80",lwd=3,lty=2)
nam<-c("Norm_mTT(AUC=0.56)","Whole_mTT(AUC=0.61)","Partial_mTT(AUC=0.56)")
legend("bottomright",nam, pch = 15,bty="n",col=colrs,cex=1,text.col=colrs[c(1,5:6)],text.font=4)


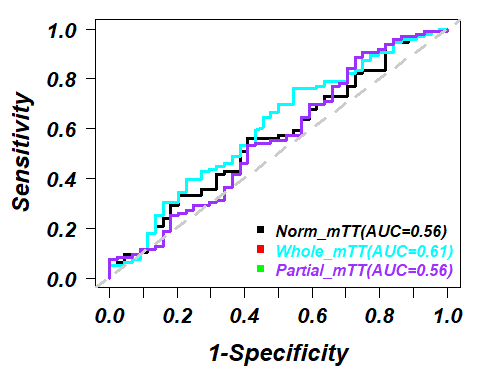


#the roc test amongst of ref_mTT, Anal_mTT and Parm_mTT
roc.test(Whole_mTT,Partial_mTT)$p.value

## [1] 0.2740797

roc.test(Whole_mTT,norm_mTT)$p.value

## [1] 0.5108524

roc.test(Partial_mTT,norm_mTT)$p.value

## [1] 0.9083326

#the comparison of sub-types of pathological results
data<-read.csv("quanti_breast_1102.csv")
#The good fitness of breast lesion with QOF>50%
cut_Whole<-which(data$Whole_QOF<50)
cut_Partial<-which(data$Partial_QOF<50)
cut<-union(cut_Whole,cut_Partial)
#remove the poor fitness
data<-data[-cut,]
#reconstruct the data only including the IDC, NPM, DCIS and fibroadenomas
fibro<-which(data$Path1=="纤维腺瘤")
gml<-which(data$Path1=="乳腺炎")
dcis<-which(data$Path1=="导管内癌")
idc<-which(data$Path1=="浸润性癌")
gml1<-which(data$Path1=="肉芽肿")
gml2<-which(data$Path1=="浆乳")
npm<-c(gml,gml1,gml2)
total<-c(fibro,dcis,idc,npm)

##whole IMAX
data_sub<-data
data_sub$Path1[fibro]<-rep("FIB",length(fibro))
data_sub$Path1[idc]<-rep("IDC",length(idc))
data_sub$Path1[dcis]<-rep("DCIS",length(dcis))
data_sub$Path1[npm]<-rep("NPM",length(npm))
data_sub<-data_sub[total,]

#The mean and standard deviation of CEUS parameters in pathological subtypes
fibro_mean<-apply(data[fibro,8:28],2,mean)
fibro_sd<-apply(data[fibro,8:28],2,sd)
cbind(fibro_mean,fibro_sd)

## fibro_mean fibro_sd
## age 43.894737 8.123319
## norm_IMAX 100.000000 0.000000
## norm_RT 12.545789 7.726639
## norm_TTP 17.310000 8.932478
## norm_mTT 61.350526 67.047958
## norm_QOF 74.231053 9.835538
## Whole_IMAX 277.974211 273.007374
## Whole_RT 9.905263 3.586646
## Whole_TTP 11.992632 5.281556
## Whole_mTT 36.756842 22.917628
## Whole_QOF 83.987895 13.569245
## Parb_IMAX 330.460000 261.715503
## Parb_RT 10.134211 5.595757
## Parb_TTP 13.683158 6.272981
## Parb_mTT 42.938421 69.449973
## Parb_QOF 72.374211 18.336607
## Partial_IMAX 406.273684 431.367195
## Partial_RT 8.311579 3.353007
## Partial_TTP 11.168421 4.365907
## Partial_mTT 26.923684 21.475274
## Partial_QOF 76.823684 14.010735

npm_mean<-apply(data[npm,8:28],2,mean)
npm_sd<-apply(data[npm,8:28],2,sd)
cbind(npm_mean,npm_sd)

## npm_mean npm_sd
## age 41.461538 14.869518
## norm_IMAX 100.000000 0.000000
## norm_RT 10.900769 8.782241
## norm_TTP 17.522308 21.237111
## norm_mTT 35.075385 46.032099
## norm_QOF 78.064615 13.295935
## Whole_IMAX 173.280769 124.163866
## Whole_RT 8.980000 4.148484
## Whole_TTP 11.250769 5.208372
## Whole_mTT 25.318462 11.738412
## Whole_QOF 82.975385 12.435444
## Parb_IMAX 210.013077 180.873070
## Parb_RT 10.481538 11.292409
## Parb_TTP 13.399231 12.994974
## Parb_mTT 29.434615 37.609913
## Parb_QOF 76.870769 11.878186
## Partial_IMAX 204.933846 142.503040
## Partial_RT 9.339231 7.419808
## Partial_TTP 14.044615 11.487160
## Partial_mTT 29.328462 30.118807
## Partial_QOF 81.760769 10.407387

dcis_mean<-apply(data[dcis,8:28],2,mean)
dcis_sd<-apply(data[dcis,8:28],2,sd)
cbind(dcis_mean,dcis_sd)

## dcis_mean dcis_sd
## age 48.833333 10.547152
## norm_IMAX 100.000000 0.000000
## norm_RT 10.973333 3.612129
## norm_TTP 15.249167 6.357597
## norm_mTT 39.162500 22.260614
## norm_QOF 75.689167 9.454230
## Whole_IMAX 198.866667 185.737606
## Whole_RT 9.604167 4.155681
## Whole_TTP 11.974167 7.181129
## Whole_mTT 34.915833 20.515870
## Whole_QOF 80.690833 15.746404
## Parb_IMAX 280.674167 451.708942
## Parb_RT 9.158333 4.185859
## Parb_TTP 12.190833 5.638905
## Parb_mTT 35.807500 28.808126
## Parb_QOF 72.856667 19.882742
## Partial_IMAX 291.011667 254.574257
## Partial_RT 8.360833 4.574869
## Partial_TTP 11.897500 7.426151
## Partial_mTT 26.976667 18.254817
## Partial_QOF 76.938333 9.975608

idc_mean<-apply(data[idc,8:28],2,mean)
idc_sd<-apply(data[idc,8:28],2,sd)
cbind(idc_mean,idc_sd)

## idc_mean idc_sd
## age 52.21250 13.269069
## norm_IMAX 100.00000 0.000000
## norm_RT 10.13175 5.840377
## norm_TTP 16.32062 7.539367
## norm_mTT 32.51863 37.248729
## norm_QOF 78.81612 9.129460
## Whole_IMAX 436.80012 501.714278
## Whole_RT 8.72550 3.015283
## Whole_TTP 11.40275 3.639696
## Whole_mTT 25.14800 17.941805
## Whole_QOF 86.36025 12.903743
## Parb_IMAX 534.04550 672.189194
## Parb_RT 8.01525 4.377925
## Parb_TTP 12.44600 5.949428
## Parb_mTT 23.59100 18.282962
## Parb_QOF 80.51825 12.889738
## Partial_IMAX 668.28125 1143.335412
## Partial_RT 7.37850 3.280736
## Partial_TTP 11.76125 4.744882
## Partial_mTT 21.82437 27.207185
## Partial_QOF 83.46775 9.250612

#p values of quantitative parameters to differentiate different histopathological subtype of breast lesions
sub_pp<-function(x){
 sub_pvalues<-rep(0,20)
 for(i in 9:28){
 lm.out<-aov(x[,i]~Path1,data=x)
 sub_pvalues[i-8]<-summary(lm.out)[[1]]$Pr[1]
}
return(sub_pvalues)
}

sub_type_p<-sub_pp(data_sub)
cbind(names(data_sub)[9:28],sub_type_p)

## sub_type_p
## [1,] "norm_IMAX" "0.136311625461905"
## [2,] "norm_RT" "0.519190470075759"
## [3,] "norm_TTP" "0.92245409561794"
## [4,] "norm_mTT" "0.0779506696946045"
## [5,] "norm_QOF" "0.269718013758014"
## [6,] "Whole_IMAX" "0.0624802532531191"
## [7,] "Whole_RT" "0.512529887605964"
## [8,] "Whole_TTP" "0.933829182457332"
## [9,] "Whole_mTT" "0.0490764960475352"
## [10,] "Whole_QOF" "0.474076541209412"
## [11,] "Parb_IMAX" "0.125745495986106"
## [12,] "Parb_RT" "0.288950141217032"
## [13,] "Parb_TTP" "0.879333851607309"
## [14,] "Parb_mTT" "0.137108701162498"
## [15,] "Parb_QOF" "0.0824235748657372"
## [16,] "Partial_IMAX" "0.236068285898452"
## [17,] "Partial_RT" "0.349854519829149"
## [18,] "Partial_TTP" "0.576570472013113"
## [19,] "Partial_mTT" "0.683530549169303"
## [20,] "Partial_QOF" "0.0305521252904279"

#In comparison of quantitative parameters of CEUS for differentitating pathological sub-types of breast lesions
par(mfrow=c(1,1))
IMAX<-data.frame(data_sub$Whole_IMAX,data_sub$Path1)
par(mar=c(3,4,1,1))
boxplot(data_sub.Whole_IMAX~data_sub.Path1,data=IMAX,col=2:5,type="l",lwd=1,xlab="",ylab="",font=4)
mtext("Maximum intensity (IMAX)",side=2,font=1,cex=1,line=2.5)


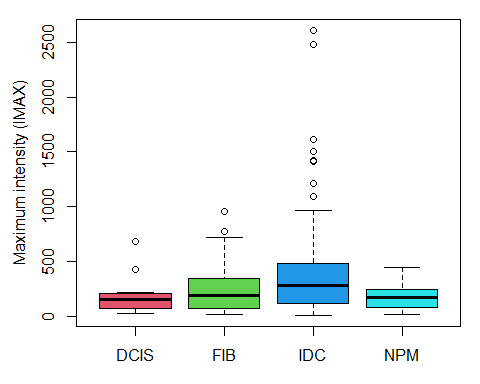


#bartlett.test(data_sub.Whole_IMAX~data_sub.Path1,data=IMAX)
#kruskal.test(data_sub.Whole_IMAX~data_sub.Path1,data=IMAX)
#pairwise.wilcox.test(data_sub$Whole_IMAX, data_sub$Path1 , p.adjust.method = "BH")

###
RT<-data.frame(data_sub$Whole_RT,data_sub$Path1)
par(mar=c(3,4,1,1))
boxplot(data_sub.Whole_RT~data_sub.Path1,data=RT,col=2:5,type="l",lwd=1,xlab="",ylab="",font=4)
mtext("Rise time (RT)",side=2,font=1,cex=1,line=2.5)


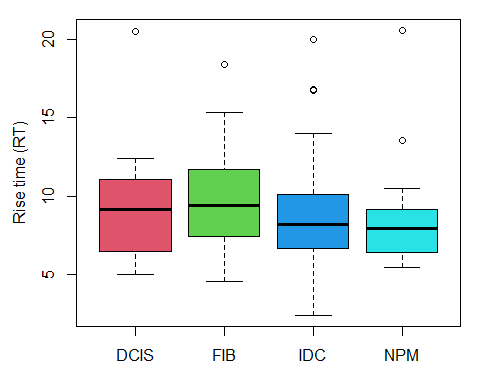


#bartlett.test(data_sub.Whole_RT~data_sub.Path1,data=RT)
#ml_RT<-aov(data_sub.Whole_RT~data_sub.Path1,data=RT)
#summary(ml_RT)[[1]]$Pr[1]
#pairwise.t.test(data_sub$Whole_RT, data_sub$Path1 , p.adjust.method = "BH")

###
TTP<-data.frame(data_sub$Whole_TTP,data_sub$Path1)
par(mar=c(3,4,1,1))
boxplot(data_sub.Whole_TTP~data_sub.Path1,data=TTP,col=2:5,type="l",lwd=1,xlab="",ylab="",font=4)
mtext("Time to peak (TTP)",side=2,font=1,cex=1,line=2.5)


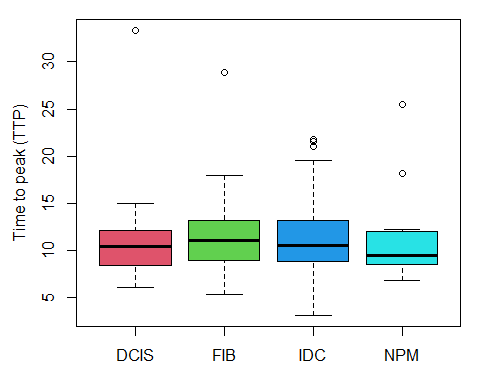


###
mTT<-data.frame(data_sub$Whole_mTT,data_sub$Path1)
par(mar=c(3,4,1,1))
boxplot(data_sub.Whole_TTP~data_sub.Path1,data=TTP,col=2:5,type="l",lwd=1,xlab="",ylab="",font=4)
mtext("Mean transit time (mTT)",side=2,font=1,cex=1,line=2.5)


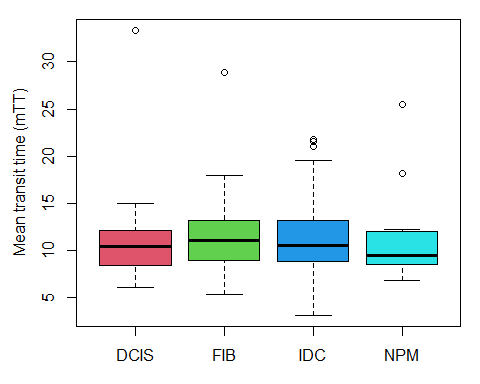


#t.test of IMAX whithin the whole lesions
t.test(data_sub$Whole_IMAX[idc],data_sub$Whole_IMAX[dcis])$p.value

## [1] 0.5778577

t.test(data_sub$Whole_IMAX[idc],data_sub$Whole_IMAX[fibro])$p.value

## [1] 0.7902046

t.test(data_sub$Whole_IMAX[idc],data_sub$Whole_IMAX[npm],)$p.value

## [1] 0.57363

t.test(data_sub$Whole_IMAX[dcis],data_sub$Whole_IMAX[fibro])$p.value

## [1] 0.7183932

t.test(data_sub$Whole_IMAX[dcis],data_sub$Whole_IMAX[npm])$p.value

## [1] 0.4144359

t.test(data_sub$Whole_IMAX[fibro],data_sub$Whole_IMAX[npm])$p.value

## [1] 0.5065842

#t.test of IMAX whithin the whole lesions
t.test(data_sub$Partial_IMAX[idc],data_sub$Partial_IMAX[dcis])$p.value

## [1] 0.3225152

t.test(data_sub$Partial_IMAX[idc],data_sub$Partial_IMAX[fibro])$p.value

## [1] 0.9068098

t.test(data_sub$Partial_IMAX[idc],data_sub$Partial_IMAX[npm])$p.value

## [1] 0.2287783

t.test(data_sub$Partial_IMAX[dcis],data_sub$Partial_IMAX[fibro])$p.value

## [1] 0.3241981

t.test(data_sub$Partial_IMAX[dcis],data_sub$Partial_IMAX[npm])$p.value

## [1] 0.1682517

t.test(data_sub$Partial_IMAX[fibro],data_sub$Partial_IMAX[npm])$p.value

## [1] 0.2374105

#t.test of IMAX whithin the whole lesions
t.test(data_sub$Whole_mTT[idc],data_sub$Whole_mTT[dcis])$p.value

## [1] 0.9263421

t.test(data_sub$Whole_mTT[idc],data_sub$Whole_mTT[fibro])$p.value

## [1] 0.1614799

t.test(data_sub$Whole_mTT[idc],data_sub$Whole_mTT[npm])$p.value

## [1] 0.3790704

t.test(data_sub$Whole_mTT[dcis],data_sub$Whole_mTT[fibro])$p.value

## [1] 0.2432823

t.test(data_sub$Whole_mTT[dcis],data_sub$Whole_mTT[npm])$p.value

## [1] 0.4351534

t.test(data_sub$Whole_mTT[fibro],data_sub$Whole_mTT[npm])$p.value

## [1] 0.947914

#t.test of IMAX whithin the whole lesions
t.test(data_sub$Partial_mTT[idc],data_sub$Partial_mTT[dcis])$p.value

## [1] 0.6052909

t.test(data_sub$Partial_mTT[idc],data_sub$Partial_mTT[fibro])$p.value

## [1] 0.4355704

t.test(data_sub$Partial_mTT[idc],data_sub$Partial_mTT[npm])$p.value

## [1] 0.9491277

t.test(data_sub$Partial_mTT[dcis],data_sub$Partial_mTT[fibro])$p.value

## [1] 0.1973252

t.test(data_sub$Partial_mTT[dcis],data_sub$Partial_mTT[npm])$p.value

## [1] 0.7106499

t.test(data_sub$Partial_mTT[fibro],data_sub$Partial_mTT[npm])$p.value

## [1] 0.4468519
