# Supplementary material for: The benefits of contrast-enhanced ultrasound in the differential diagnosis of suspicious breast lesions
Source: Front Med (Lausanne). 2024 Dec 24;11:1511200. doi: 10.3389/fmed.2024.1511200 (PMC11703730; doi:10.3389/fmed.2024.1511200)
Supplement: Supplementary file 2 [file SM_Table_1_1511200.docx]

**Supplementary Table 1.** Features of the patients and their ultrasonic features of suspicious breast lesions

| **Type of feature** | **Benign(n=49）** | **Malignant(n=101）** | ***P* value** |
| --- | --- | --- | --- |
| **Age (year)** | 44.6±10.9 | 52.8±13.5 | <0.01^c^ |
| **Familial inheritance**  **No**  **Yes** | 46  3 | 82  19 | 0.07 ^a^ |
| **Menostasia history**  **No**  **Yes** | 36  12 | 49  52 | <0.01 ^a^ |
| **Location**  **Upper outer**  **Lower outer**  **Upper Inter**  **Lower Inter**  **Nipple** | 22  13  9  4  1 | 47  24  14  15  1 | <0.25 ^b^ |
| **The distance to nipple (cm)** | 1.9±1.3 | 2.5±1.8 | 0.048 ^c^ |
| **Size (mm)** | 18.2±12.1 | 23.2±13.3 | 0.03 ^c^ |
| **Shape**  **Regular**  **Irregular** | 21  28 | 21  80 | 0.01 ^a^ |
| **Margin**  **Clear**  **Unclear** | 37  12 | 32  69 | <0.01 ^a^ |
| **Calcification**  **None**  **Microcalcificatons**  **Macrocalcifications** | 43  5  1 | 63  16  22 | <0.01 ^a^ |
| **Blood vessels (No.)**  **0**  **1**  **2**  **3** | 11  21  8  9 | 0  23  38  40 | <0.01 ^b^ |
| **Axillary lymph nodes**  **No**  **Yes** | 49  0 | 53  48 | <0.01 ^a^ |
| **Enlarged scope**  **NO**  **YES** | 36  13 | 37  64 | <0.01 ^a^ |
| **Irregular shape**  **NO**  **YES** | 28  21 | 33  68 | <0.01 ^a^ |
| **Directed perfusion**  **NO**  **YES** | 27  22 | 59  42 | 0.70 |
| **Heterogeneous perfusion**  **NO**  **YES** | 29  20 | 20  81 | <0.01 ^a^ |
| **Perfusion deficiency**  **NO**  **YES** | 29  20 | 23  78 | <0.01 ^a^ |
| **Crab clam-like enhancement**  **NO**  **YES** | 47  2 | 39  62 | <0.01 ^a^ |
| **More than two enhanced vessels within lesions**  **NO**  **YES** | 44  5 | 40  61 | <0.01 ^a^ |
| **Surrounding enriched vessels with inserting into lesions**  **NO**  **YES** | 30  19 | 0  101 | <0.01 ^a^ |

**^a^ Chi-square test, ^b^ Fisher’s exact test, ^c^ t-test.**
