# Supplementary material for: The benefits of contrast-enhanced ultrasound in the differential diagnosis of suspicious breast lesions
Source: Front Med (Lausanne). 2024 Dec 24;11:1511200. doi: 10.3389/fmed.2024.1511200 (PMC11703730; doi:10.3389/fmed.2024.1511200)
Supplement: Supplementary file 3 [file SM_Table_2_1511200.docx]

**Supplementary Table 2.** In comparison of quantitative parameters of CEUS for differentiating the atypical benign from malignant breast lesions

| **Quantitative parameters** | **Benign**  **(n=44)** | **Malignant**  **( n=96 )** | **P value**  **( t-test )** |
| --- | --- | --- | --- |
| **Norm_ IMAX(%)** | 100±0 | 100±0 | 1 |
| **Norm _ RT(s)** | 10.1±7.2 | 10.2±5.6 | 0.50 |
| **Norm _ TTP(s)** | 16.6±13.0 | 16.3±7.6 | 0.91 |
| **Norm _ mTT(s)** | 45.7±52.9 | 33.5±35.5 | 0.17 |
| **Whole_ IMAX(%)** | 226.8±210.4 | 411.3±482.9 | <0.01 |
| **Whole _ RT(s)** | 10.1±4.9 | 8.8±3.2 | 0.13 |
| **Whole _ TTP(s)** | 12.4±5.9 | 11.5±4.2 | 0.33 |
| **Whole mTT(s)** | 38.6±51.9 | 26.3±18.3 | 0.13 |
| **Partial_ IMAX(%)** | 329.7±325.6 | 629.0±1074.1 | 0.01 |
| **Partial _ RT(s)** | 8.4±4.9 | 7.5±3.4 | 0.32 |
| **Partial _ TTP(s)** | 12.9±8.1 | 11.8±5.0 | 0.37 |
| **Partial _ mTT(s)** | 27.2±23.6 | 22.4±25.7 | 0.28 |

IMAX, maximum intensity; RT, rising time; TTP, time to peak; mTT, mean transit time.
