# Supplementary material for: The benefits of contrast-enhanced ultrasound in the differential diagnosis of suspicious breast lesions
Source: Front Med (Lausanne). 2024 Dec 24;11:1511200. doi: 10.3389/fmed.2024.1511200 (PMC11703730; doi:10.3389/fmed.2024.1511200)
Supplement: Supplementary file 4 [file SM_Table_3_1511200.docx]

**Supplementary Table 3.** In comparison of qualitative risk features of CEUS for differentiating histopathological sub-types of suspicious breast lesions

| **Risk features** | **IDC**  **(n=83)** | **DCIS**  **(n=12)** | **Atypical FIB**  **(n=20)** | **Mass-like NPM**  **(n=14)** | **P value**  **(Chi-square)** |
| --- | --- | --- | --- | --- | --- |
| **Enlarged scope (%)** | 55 (66.3) | 6 (50.0) | 2 (10.0) | 10 (71.4) | <0.01 |
| **Irregular shape (%)** | 56 (67.5) | 9 (75.0) | 3 (15.0) | 10 (71.4) | <0.01 |
| **Directed perfusion (%)** | 40 (48.2) | 9 (75.0) | 7 (35.0) | 9 (64.3) | 0.11 |
| **Heterogeneous perfusion (%)** | 68 (81.9) | 9 (75.0) | 7 (35.0) | 8 (57.1) | <0.01 |
| **Perfusion deficiency (%)** | 66 (79.5) | 8 (66.7) | 5 (25.0) | 11 (78.6) | <0.01 |
| **Crab clam-like enhancement (%)** | 52 (62.7) | 6 (50.0) | 1 (5.0) | 1 (7.1) | <0.01 |
| **More than two enhanced vessels within lesions (%)** | 50 (60.2) | 7 (58.3) | 2 (10.0) | 2 (14.3) | <0.01 |
| **Surrounding enriched vessels with inserting into lesions (%)** | 67 (80.7) | 11 (91.7) | 14 (10.0) | 11 (42.9) | <0.01 |

IDC, invasive ductal carcinoma; DCIS, ductal carcinoma in site; FIB, fibroadenoma; NPM, non-puerperal mastitis.
