# Supplementary material for: The benefits of contrast-enhanced ultrasound in the differential diagnosis of suspicious breast lesions
Source: Front Med (Lausanne). 2024 Dec 24;11:1511200. doi: 10.3389/fmed.2024.1511200 (PMC11703730; doi:10.3389/fmed.2024.1511200)
Supplement: Supplementary file 5 [file SM_Table_4_1511200.docx]

**Supplementary Table 4.** The distribution of quantitative parameters of CEUS between malignant and atypical benign breast lesions

| **Quantitative**  **Parameters** | **IDC**  **(n=80)** | **DCIS**  **(n=12)** | **Atypical FIB**  **(n=19)** | **Mass-like NPM**  **(n=13)** | **P value**  **(ANOVA)** |
| --- | --- | --- | --- | --- | --- |
| **Norm _ IMAX (%)** | 100±0 | 100±0 | 100+0 | 100+0 | / |
| **Norm _ RT (s)** | 10.1±5.8 | 11.0±3.6 | 12.5±7.7 | 10.9±8.9 | 0.51 |
| **Norm _ TTP (s)** | 16.3±7.5 | 15.6±6.4 | 17.3±8.9 | 17.5±21.2 | 0.92 |
| **Norm _ mTT (s)** | 32.5±37.3 | 39.2±22.3 | 61.4±67.0 | 35.1±46.0 | 0.08 |
| **Whole _ IMAX (%)** | 534.1±672.2 | 198.9±185.7 | 278.0±273.0 | 173.3±124.2 | 0.06 |
| **Whole _ RT (s)** | 8.7±3.0 | 9.6±4.7 | 9.9±3.6 | 9.0±4.2 | 0.51 |
| **Whole _ TTP (s)** | 11.4±3.6 | 12.0±7.2 | 12.0±5.3 | 11.3±11.2 | 0.93 |
| **Whole _ mTT (s)** | 25.2±17.9 | 34.9±20.5 | 36.8±22.9 | 25.3±11.7 | 0.05 |
| **Partial _ IMAX (%)** | 668.3±1143.3 | 291.0±254.6 | 406.3±431.4 | 204.9±142.5 | 0.24 |
| **Partial _ RT (s)** | 7.4±3.3 | 8.4±4.6 | 8.3±3.4 | 9.3±7.4 | 0.35 |
| **Partial _ TTP (s)** | 11.8±4.7 | 11.9±7.4 | 11.2±4.4 | 14.0±11.5 | 0.58 |
| **Partial _ mTT (s)** | 21.8±27.2 | 27.0±18.3 | 26.9±21.5 | 29.3±30.1 | 0.68 |

IDC, invasive ductal carcinoma; DCIS, ductal carcinoma in site; FIB, fibroadenoma; NPM, non-puerperal mastitis; IMAX, maximum intensity; RT, rising time; TTP, time to peak; mTT, mean transit time.
