# Supplementary material for: The benefits of contrast-enhanced ultrasound in the differential diagnosis of suspicious breast lesions
Source: Front Med (Lausanne). 2024 Dec 24;11:1511200. doi: 10.3389/fmed.2024.1511200 (PMC11703730; doi:10.3389/fmed.2024.1511200)
Supplement: Supplementary file 6 [file SM_Table_5_1511200.docx]

**Supplementary table 5. Diagnostic efficacy of different risk characteristics and integrated models between of suspicious breast lesions**

**Diagnostic efficacy of different risk characteristics and integrated models between Atypical FIB and Mass-like NPM**

|  | **Sensitivity** | **Specificity** | **AUC** | **95%CI** |
| --- | --- | --- | --- | --- |
|  |  |  |  |  |
| **Heterogeneous perfusion model** | 0.53 | 0.69 | 0.61 | 0.43-0.77 |
| **Crab clam-like enhancement model** | 0.07 | 0.95 | 0.51 | 0.33-0.69 |
| **Partial_ IMAX model** | 0.50 | 0.99 | 0.73 | 0.56-0.90 |
| **Integrated model** | 0.43 | 0.95 | 0.69 | 0.50-0.87 |

**Diagnostic efficacy of different risk characteristics and integrated models between IDC and DCIS**

|  | **Sensitivity** | **Specificity** | **AUC** | **95%CI** |
| --- | --- | --- | --- | --- |
| **Heterogeneous perfusion model** | 0.82 | 0.25 | 0.54 | 0.35-0.72 |
| **Crab clam-like enhancement model** | 0.63 | 0.50 | 0.56 | 0.39-0.74 |
| **Partial_ IMAX model** | 0.57 | 0.58 | 0.52 | 0.33-0.71 |
| **Integrated model** | 0.82 | 0.50 | 0.58 | 0.38-0.78 |

**Diagnostic efficacy of different risk characteristics and integrated models between benign and malignant lesions**

|  | **Sensitivity** | **Specificity** | **AUC** | **95%CI** |
| --- | --- | --- | --- | --- |
| **Heterogeneous perfusion model** | 0.80 | 0.59 | 0.70 | 0.62-0.77 |
| **Crab clam-like enhancement model** | 0.61 | 0.95 | 0.78 | 0.71-0.85 |
| **Partial_ IMAX model** | 0.42 | 0.78 | 0.60 | 0.52-0.68 |
| **Integrated model** | 0.83 | 0.78 | 0.89 | 0.83-0.94 |
