# Supplementary figures and images for: The benefits of contrast-enhanced ultrasound in the differential diagnosis of suspicious breast lesions
Source: Front Med (Lausanne). 2024 Dec 24;11:1511200. doi: 10.3389/fmed.2024.1511200 (PMC11703730; doi:10.3389/fmed.2024.1511200)

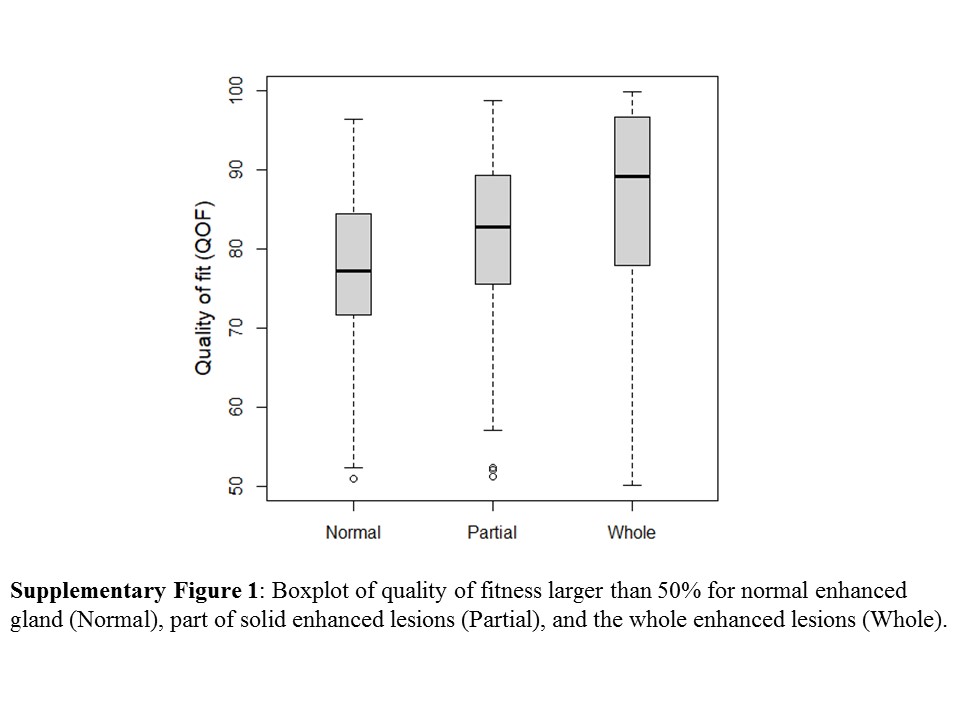

Supplement: Supplementary file 7 [file Image_1.JPEG]

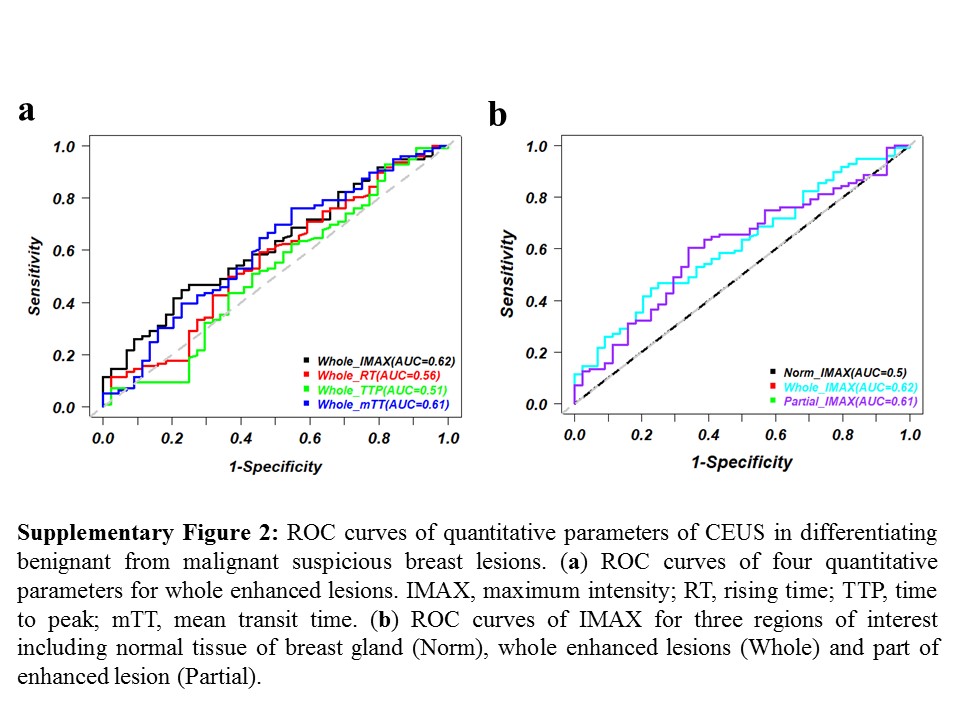

Supplement: Supplementary file 8 [file Image_2.JPEG]

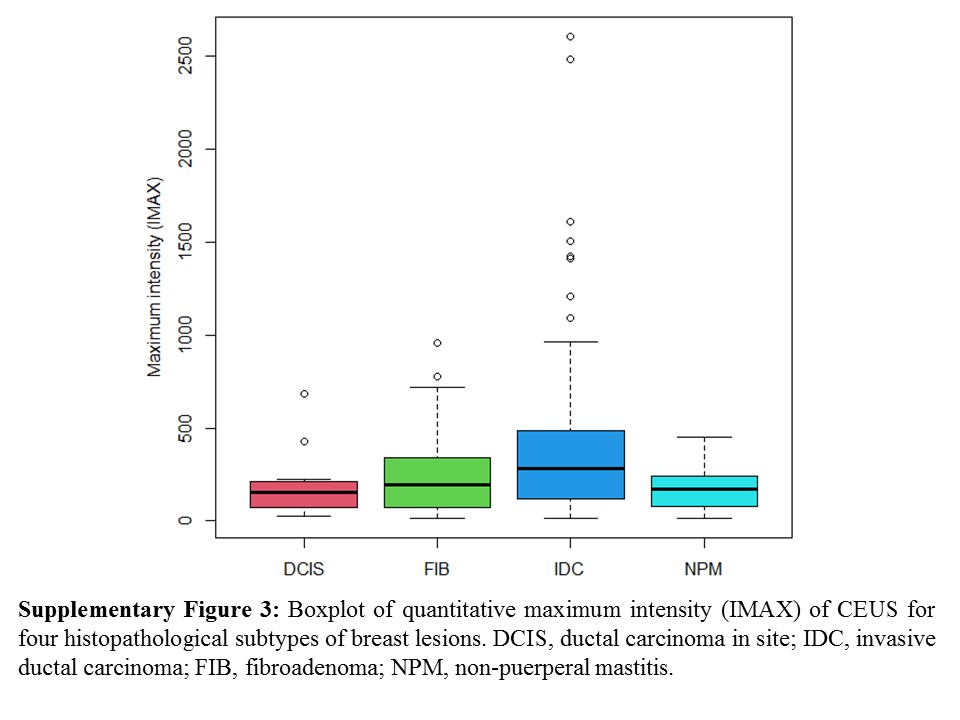

Supplement: Supplementary file 9 [file Image_3.JPEG]
